# Antibody-drug conjugates in cancer therapy: current advances and prospects for breakthroughs

**DOI:** 10.3389/fcell.2025.1669592

**Published:** 2025-10-08

**Authors:** Dan Wu, Kaixuan Yang, Runjia He, Rutie Yin, Lin Shui

**Affiliations:** 1 Cancer Center, West China Second University Hospital, Sichuan University, Chengdu, Sichuan, China; 2 Key Laboratory of Birth Defects and Related Diseases of Women and Children, Sichuan University, Chengdu, Sichuan, China; 3 School of Pharmacy, Southwest Medical University, Luzhou, Sichuan, China; 4 Acupuncture and Tuina School, Chengdu University of Traditional Chinese Medicine, Chengdu, Sichuan, China

**Keywords:** antibody drug conjugate (ADC), antitumor treatment, linker, payload, drug approval

## Abstract

Antibody-drug conjugates (ADCs), often referred to as “intelligent biological missiles,” have garnered significant attention in the rapidly evolving landscape of cancer therapy. ADCs represent a sophisticated approach by integrating monoclonal antibodies (mAbs), which are particular targeting tumor antigens, with cytotoxic payloads, which deliver lethal effects. Compared with the combination of chemotherapy and mAbs, ADCs precisely deliver highly potent cytotoxins directly to tumor cells while minimizing damage to healthy tissues. However, limitations such as significant adverse effects, suboptimal therapeutic efficacy, and drug resistance require carefully evaluation and further optimization. Further studies are necessary to explore the next-generation of ADCs, such as the combination of ADCs with other anti-tumor strategies, bispecific ADCs, dual-payload ADCs and radionuclide drug conjugates (RDCs). This review provides a comprehensive overview of recent developments in oncology treatment, focusing on the historical evolution, structural design, clinical advancements, and mechanisms of action of approved ADCs. Each structural element, including the target antigen, mAb, linker system, and cytotoxic payload, as well as advancements in payload conjugation technology, plays a critical role in the development of ADCs. Through ongoing refinement and innovation, it is anticipated that next-generation ADCs with enhanced therapeutic benefits for patient populations can be realized.

## Graphical Abstract

**Figure:**
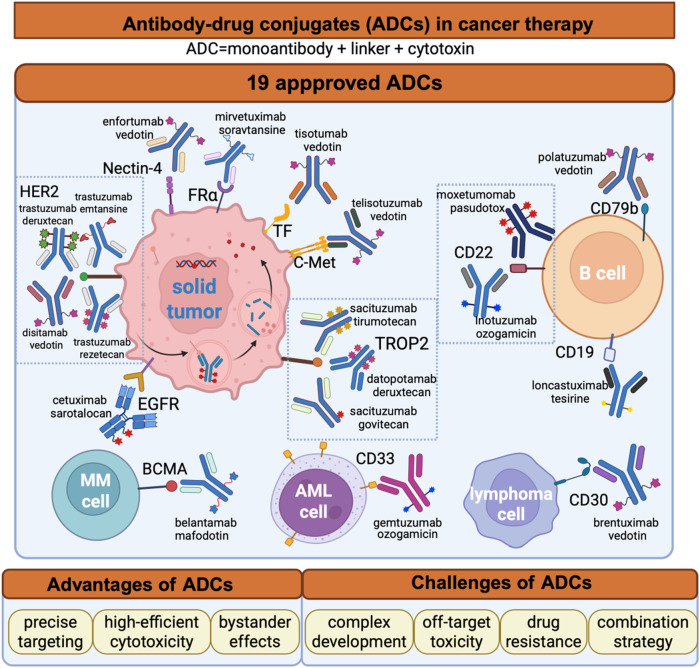

## 1 Introduction

Cancer continues to be a major disease that poses a significant threat to human health and life (Rebecca et al., 2025). While traditional cancer treatments, including surgery, chemotherapy, and radiation therapy, can partially inhibit tumor growth, each approach is associated with notable limitations (DeVita and Rosenberg, 2012). Consequently, there is an urgent demand for novel therapeutic agents to improve the efficacy of cancer treatment. Antibody-drug conjugates (ADCs) represent sophisticated constructs that combine a cytotoxic payload with a mAb, harnessing the target specificity with the potent cytotocixity, gaining increasing attention in the fight against cancer.

**FIGURE 1:**
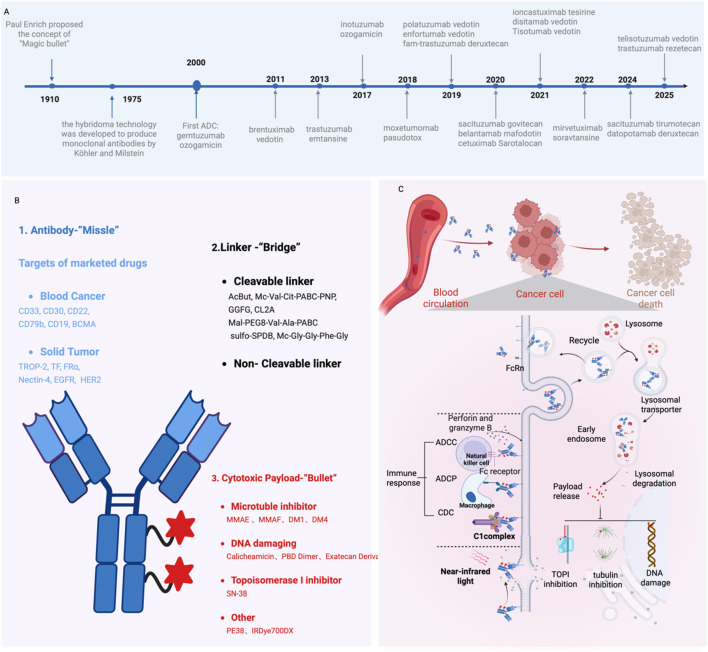
The Development and Mechanism of ADCs in Oncology Treatment. **(A)** Timeline of key milestones in ADC development over the past century; **(B)** Structural composition of ADCs; **(C)** Mechanism of action for ADC-mediated cancer cell elimination. Figure was created by Biorender.com.

The year 2000 marked a pivotal milestone in the development of ADCs. Gemtuzumab ozogamicin received approval from the U.S Food and Drug Administration (FDA) for treating CD33-positive acute myeloid leukemia (AML), establishing it as the first commercially available ADC (Bross et al., 2001; Linenberger et al., 2001). However, due to suboptimal efficacy and severe side effects, Gemtuzumab ozogamicin was voluntarily withdrawn from the market in 2010 (Petersdorf et al., 2013; Ricart, 2011). Despite this setback, ADC research continued to progress, leading the advancement of second-generation ADCs. In 2011, brentuximab vedotin received approval for the treatment of Hodgkin lymphoma (HL) and systemic anaplastic large cell lymphoma (sALCL) (Katz et al., 2011). In 2013, trastuzumab emtansine became the first ADC approved in the treatment for solid tumors (Ballantyne and Dhillon, 2013). More recently, the approval of third-generation ADCs, including trastuzumab deruxtecan and sacituzumab govitecan achieved remarkable clinical results (Lambert and Chari, 2014; Syed, 2020). As of June 2025, a total of 19 ADCs have been approved globally for the treatment of various hematological malignancies and solid tumors (Figure 1A; Table 1). However, comprehensive and up-to-date summary regarding globally approved ADCs and emerging ADC candidates remains limited. This review provides a comprehensive overview of recent research advances in antitumor ADCs and a critical discussion of current limitations and emerging future directions in ADC development.

**TABLE 1:** Detailed information about 19 approved ADC drugs as of June 2025.

No.	Name	Target	Linker	Payload	DAR	Indication first approved	First approval
1	Gemtuzumab ozogamicin (Mylotarg®)	CD33	AcBut	Calicheamicin derivative (ozogamicin)	2–3	CD33-positive R/R AML	5/2000 FDA
2	Brentuximab vedotin (Adcetris®)	CD30	Mc-Val-Cit-PABC	MMAE	4	CD30-positive R/R HL and sALCL	8/2011 FDA
3	Trastuzumab emtansine (Kadcyla®)	HER2	Non-cleavable linker	DM1	3.5	HER2-positive mBC patients who previously treated with trastuzumab and taxane-based chemotherapy	2/2013 FDA
4	Inotuzumab ozogamicin (Besponsa®)	CD22	AcBut	ozogamicin	6	adults patients with R/R B-ALL	5/2017 FDA
5	Moxetumomab pasudotox (Lumoxiti®)	CD22	Mc-Val-Cit-PABC	PE38	—	adult patients with R/R HCL	9/2018 FDA
6	Polatuzumab vedotin (Polivy®)	CD79b	Mc-Val-Cit-PABC	MMAE	3–4	combine with bendamustine and rituximab for patients with R/R DLBCL who have undergone at least two prior therapies	1/2019 FDA
7	Enfortumab vedotin (Padcev®)	Nectin-4	Mc-Val-Cit-PABC	MMAE	3.8	la/m UC	12/2019 FDA
8	Trastuzumab deruxtecan (Enhertu®)	HER2	glycyl-glycyl-phenylalanyl-glycyl (GGFG)	DXd	8	la/m HER2-positive BC who had already received two or more anti-HER2 therapies	12/2019 FDA
9	Sacituzumab govitecan (Trodelvy®)	TROP-2	CL2A	SN38	7.6	la/m TNBC who have undergone at least two prior systemic treatments	4/2020 FDA
10	Belantamab mafodotin (Blenrep®)	BCMA	Non-cleavable linkers	MMAF	4	R/R MM	8/2020 FDA
11	Cetuximab Sarotalocan (Akalux®)	EGFR	Non-cleavable linkers	IRDye700	1.3–3.8	la/m HNSCC	9/2020 FDA
12	Loncastuximab tesirine (Zynlonta®)	CD19	Mal-PEG8-Val-Ala-PABC	PBD dimer (SG3199)	2.3	adult patients with R/R LBL after receiving two or more systemic therapies	4/2021 FDA
13	Disitamab vedotin (Aidixi®)	HER2	Mc-Val-Cit	MMAE	4	la/m GC/GEJC who had undergone at least two lines of systemic chemotherapy	6/2021 NMPA
14	Tisotumab vedotin (Tivdak®)	TF	Mc-Val-Cit-PABC	MMAE	4	recurrent or mCC who progressed after chemotherapy	9/2021 FDA
15	Mirvetuximab soravtansine (Elahere®)	FR-α	sulfo-SPDB	DM4	3–4	FRα-positive PROC in patients who had undergone less than 3 lines of therapies	11/2022 FDA
16	Sacituzumab tirumotecan (佳泰莱®)	TROP-2	CL2A	Top I inhibitors (KL610023)	7.4	la/m TNBC who have undergone at least two prior systemic therapies	11/2024 NMPA
17	Datopotamab deruxtecan (Datroway®)	TROP-2	GGFG	DXd	4	HR^+^HER2^-^ la/mBC who have previously been treated with endocrine therapy and chemotherapy	12/2024 PMDA
18	Telisotuzumab vedotin (Emrelis®)	c-Met	Mc-Val-Cit-PABC	MMAE	3	la/m NSCLC with high overexpression of the c-Met after prior systemic treatment	5/2025 FDA
19	Trastuzumab rezetecan (SHRA 1811®)	HER2	Mc-Gly-Gly-Phe-Gly	Top I inhibitors (SHR169265)	5.7	advanced HER2-mutant NSCLC	5/2025 NMPA

Abbreviation: FDA, U.S., food and drug administration; NMPA, china national medical products administration; PMDA, the Pharmaceuticals and Medical Devices Agency; R/R, relapsed/refractor; AML, acute myeloid leukemi; HL, hodgkin lymphoma; sALCL, systemic anaplastic large cell lymphoma; HER2, human epidermal growth factor receptor 2; mBC, metastatic breast cancer; B-ALL,B-cellacute lymphoblastic leukemia; HCL, hairy cell leukemia; DLBCL, diffuse large B-cell lymphoma; UC, urothelial carcinoma; TNBC, triple-negative breast cancer; MM, multiple myeloma; NSCLC, non-small cell lung cancer; LBL, lymphoblastic lymphoma; GC/GEJC, gastric cancer/gastroesophageal junction cancer; CC, cervical cancer; PROC, platinum-resistant ovarian cancer; Nectin-4, Nectin cell adhesion molecule 4; BCMA, B-Cell maturation antigen; EGFR, epidermal growth factor receptor; c-Met, cellular-mesenchymal epithelial transition factor; Trop-2, Trophoblast cell-surface antigen-2; FRα, folate receptor alpha; TF, tissue factor.

## 2 Core components of ADCs

Generally, ADCs consist of three critical components: a mAb, a cytotoxic payload, and a chemical linker (Figure 1B). Each of these elements plays an indispensable role in determining the efficacy and safety profile of ADCs.

### 2.1 Targeting module--monoclonal antibody (mAb)

MAbs function as the targeting component in ADCs, enabling the specific recognition and binding to antigens expressed on the surface of cancer cells. To achieve optimal performance, mAbs need to exhibit low immunogenicity, high specificity and affinity for the target antigen, an extended half-life, and stability during circulation in the bloodstream (Köhler and Milstein, 1975; Goldmacher and Kovtun, 2011). Humanized or fully human mAbs are favored due to the high specificity for cell targeting, extended circulation time in human blood, and diminished immunogenicity (Tsuchikama and An, 2016). Due to its sustained efficacy, adaptability for engineering, and well-established conjugation methods, IgG has become the preferred choice for ADCs. Additionally, IgG demonstrates potent effector functions mediated by its Fc segment (Hoffmann et al., 2018), such as antibody-dependent cell-mediated cytotoxicity (ADCC) and complement-dependent cytotoxicity (CDC). Besides IgG antibodies, innovative antibody formats, such as single-chain fragment variables (scFvs) and nanobodies have gained increasing attention in the development of ADCs.

### 2.2 Therapeutic payload--cytotoxin

In most ADCs, payloads function as the key mediators of cytotoxic activity. First-generation ADCs predominantly used traditional chemotherapeutic agents, such as methotrexate, vinblastine, and doxorubicin, as cytotoxic payloads. However, due to insufficient cytotoxicity against cancer cells and limited accumulation in target cells, the efficacy of these early ADCs was inferior even to the parent compounds, leading to clinical failure. Subsequently, researchers focused on the discovery of novel and highly potent cytotoxic compounds derived from natural sources, such as plants and microorganisms, which exhibit anti-tumor activity that is 100–1000 times stronger compared to conventional chemotherapeutic agents. Historically, early clinical trials identified significant side effects due to its non-specific high toxicity. Subsequently, medicinal chemists synthetically modified the cytotoxins, resulting in the development of its derivatives for use as payloads in ADCs. A wide range of cytotoxins are commonly employed in research, such as tubulin inhibitors, DNA-damaging agents, and others (Conilh et al., 2023).

#### 2.2.1 Tubulin inhibitors

**FIGURE 2:**
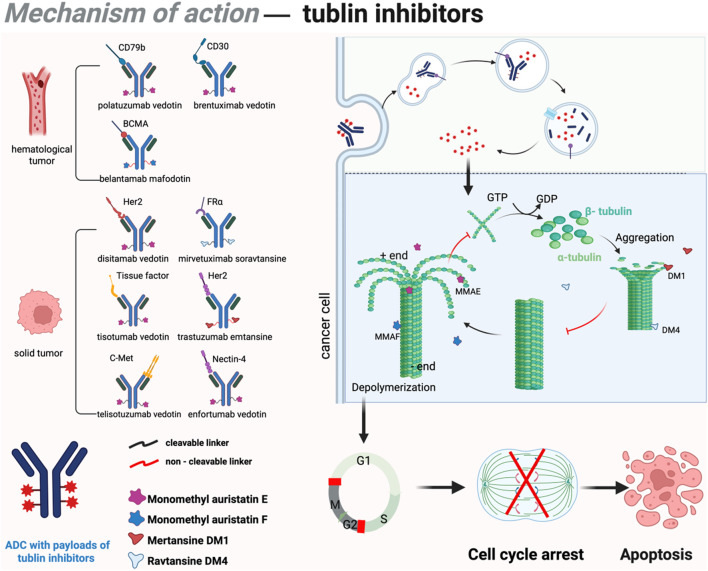
Mechanism of action of tubulin inhibitors in ADCs. DM1 and DM4 bind to β-tubulin, promoting polymerization and inhibiting depolymerization, leading to abnormal microtubule stabilization, mitotic spindle disruption, chromosome segregation errors, and G2/M phase arrest. This triggers apoptosis via the mitochondria-dependent pathway. Conversely, MMAE and MMAF bind to α/β-tubulin, suppressing polymerization and accelerating disassembly, reducing microtubule density, impairing spindle assembly, and causing prophase/metaphase arrest. These agents also induce apoptosis indirectly through oxidative stress and mitochondrial membrane potential loss. Figure was created by Biorender.com.

Most second-generation ADCs incorporate tubulin inhibitors as payloads. Microtubules function as critical cytoskeletal components, playing a pivotal role in intracellular transport, cell division, cellular motility, and the maintenance of cellular morphology (Hohmann and Dehghani, 2019). Specifically, the entire microtubule network rearranges to form the mitotic spindle during cell division, thus providing the structural framework for the physical segregation of sister chromatids. Tubulin inhibitors disrupts the dynamic equilibrium of microtubule assembly and disassembly, arresting cells in the G2/M phase of the cell cycle and ultimately triggering apoptosis (Janke and Magiera, 2020). Detailed mechanism and approved drugs are listed in Figure 2. Common payloads used in ADCs such as auristatin and maytansine. Among auristatin derivatives, monomethyl auristatin E (MMAE) and monomethyl auristatin F (MMAF) are the most widely adopted in ADC research. Unlike MMAE, MMAF is more hydrophilic, less prone to aggregation, and exhibits lower systemic toxicity (Markham, 2020). To date, seven approved ADC drugs utilize MMAE or MMAF as payloads, accounting for more than 50% of all ADCs under development. DM1 and DM4 are the two most commonly employed maytansine derivatives in clinical practice. Currently, approximately 20% of ADCs in development incorporate maytansine derivatives as the cytotoxic payload (Xi et al., 2024).

#### 2.2.2 DNA-damaging agents

**FIGURE 3:**
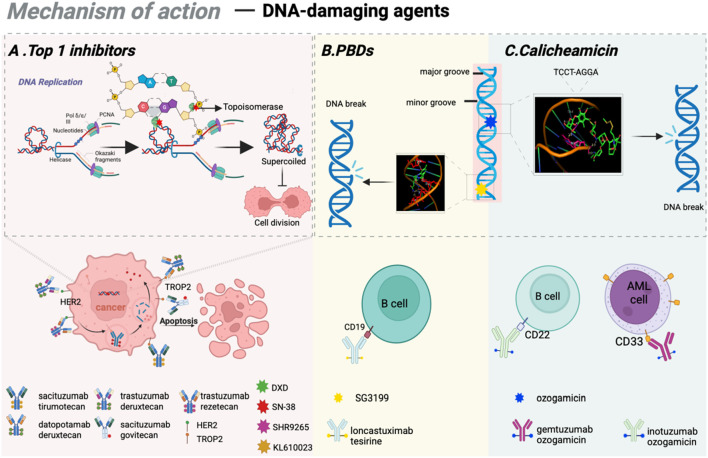
Mechanisms of action of DNA-damaging agents. **(A)** The mode of action of Top 1 inhibitors and the clinically approved ADCs based on Top 1 inhibitos; **(B)** The working mechanism of PBDs and clinically approved ADCs based on PBDs; **(C)** The mechanism of calicheamicin as DNA-damaging agents. Figure was created by Biorender.com.

Although tubulin inhibitors demonstrate high efficacy against rapidly proliferating tumor cells, their activity is significantly diminished in quiescent cancer cell populations. To overcome this limitation, third-generation ADCs primarily utilize DNA-damaging agents capable of targeting all phases of the cell cycle as cytotoxic payloads. These agents disrupt DNA structure through mechanisms such as double-strand breaks, alkylation, intercalation, and cross-linking (Roos and Kaina, 2013; Brinkman et al., 2021). Notable examples of DNA inhibitors encompass topoisomerase I (Top1) inhibitors, calicheamicin, and the pyrrolobenzodiazepines (PBDs). In recent years, 8 ADCs based on DNA-damaging agents are approved for anti-tumor treatment in clinical practice. Detailed mechanisms and approved drugs are listed in Figure 3.

##### 2.2.2.1 Top 1 inhibitors

Topoisomerases are nuclear enzymes that play an essential role in resolving DNA supercoiling, thereby facilitating critical cellular processes such as transcription and replication (Goffart et al., 2019). Top1 inhibitors function by forming DNA-topoisomerase complexes, which suppress enzyme activity and induce DNA double-strand breaks, disrupting DNA replication and transcription in tumor cells, ultimately leading to cell death (Akaiwa et al., 2020; Hartmann et al., 2020). By establishing stable complexes with Top 1, camptothecin obstructs the typical interaction between topoisomerases and DNA, thus suppressing DNA replication and transcription. SN-38 is the principal active metabolite of Top 1 inhibitor (irinotecan) and demonstrates 100 to 1000 times greater anti-tumor activity. Dxd, a derivative of novel Top 1 inhibitor (exatecan), shows approximately 10 times greater potency than SN-38. Moreover, Dxd exhibits reduced bone marrow toxicity, offering a significant safety advantage compared to other agents. Numerous ADC drugs featuring Dxd as the payload are currently in various stages of clinical trials.

##### 2.2.2.2 Calicheamicin

Calicheamicin represents a class of DNA-targeting anti-tumor antibiotics derived from the fermentation product of *Streptomyces* platensis (Lee et al., 1991). As one of the most toxic natural compounds, calicheamicin binds selectively to the TCCT-AGGA sequence located within the minor groove of the DNA double helix, leading to DNA strand scission and ultimately inducing apoptosis in tumor cells (Ben-Zvi et al., 2024). To date, FDA-approved ADC drugs such as Gemtuzumab ozogamicin and inotuzumab ozogamicin (Besponsa) utilize calicheamicin as cytotoxic agents.

##### 2.2.2.3 PBDs

PBD, a class of antibacterial and anti-tumor compounds discovered in the 1960s, initially existed as monomers that bind specifically to minor grooves of DNA sequences (Mantaj et al., 2017). This interaction stabilizes the helical structure of DNA, thereby inhibiting the cell division process, inducing cell cycle arrest at the G2/M phase, and ultimately resulting in apoptosis. PBD dimers show considerable promise as payloads due to the ability to create interstrand cross-links while causing minimal structural changes in DNA, therefore effectively bypassing DNA repair pathways (Wells et al., 2006; Rettig et al., 2009). The latest FDA-approved ADC drug, loncastuximab tesirine, utilizes PBD dimers as cytotoxic payloads.

### 2.3 Conjugation bridge--linker

As a critical component in ADCs, the linker serves as the “bridge” by securely connecting the antibody to the cytotoxin. A fundamental requirement for linkers is to maintain chemical stability within the bloodstream while enabling rapid and efficient payload release at the target site following cellular internalization (Aoyama et al., 2024; Lucas et al., 2018). Diverse types of linkers can generally be classified into cleavable and non-cleavable categories (Tsuchikama and An, 2016; Bargh et al., 2019).

#### 2.3.1 Cleavable linkers

Cleavable linkers constitute a major category employed in ADC development. In contrast to non-cleavable linkers, cleavable linkers facilitate drug release within target cells under specific intracellular conditions, such as acidic environments, reducible conditions, specific enzymes and external stimuli (e.g., photons). Although cleavable linkers generally exhibit lower systemic stability and carry a higher risk of off-target toxicity, they offer greater versatility and can be paired with a broader range of payloads.

#### 2.3.2 Non-cleavable linkers

Non-cleavable linkers are composed of highly stable chemical bonds that resist proteolytic degradation, thereby providing superior stability compared to cleavable counterparts. ADCs utilizing non-cleavable linkers depend on complete lysosomal degradation of the antibody for payload release, which results in concurrent linker breakdown. However, due to the lack of membrane permeability in the charged residual moiety, non-cleavable linkers are generally incapable of inducing bystander effects, which significantly restricts their application potential. To date, only two ADCs incorporating non-cleavable linkers have received regulatory approval.

### 2.4 Drug-antibody ratio (DAR)

The DAR is a critical parameter used to quantify the average number of cytotoxic payloads conjugated to each antibody molecule in ADCs, which influences both the therapeutic efficacy and safety profile. Specifically, a suboptimal DAR value may diminish the anti-tumor effects, whereas an excessively high DAR can compromise antibody structure and stability, potentially leading to reduced pharmacological activity.

## 3 Mechanism of ADCs

ADCs are a class of large molecular that undergo cellular internalization through membrane encapsulation, forming vesicular structures that facilitate transport into the cytoplasm-a process called endocytosis. Specifically, ADCs selectively bind to tumor antigen and form ADC-antigen complexes, subsequently internalized into the cell through clathrin- or caveolin-mediated endocytosis and enclosed in early endosomes, which progressively mature into late endosomes before fusing with lysosomes (Fu et al., 2022). Within the lysosomal compartment, ADCs are fully degraded by cathepsins through a series of complex proteolytic processes. The released cytotoxic payloads are then transported from lysosomes into the cytoplasm, where they exert the therapeutic effects to induce cell apoptosis. Beyond the direct cytotoxic effects, certain ADCs possess the ability to cross cellular membranes and affect adjacent cells (de Bever et al., 2023). This phenomenon, known as the bystander effect, significantly enhances the therapeutic potential of ADCs and typically requires cleavable linkers to enable efficient payload release (Staudacher and Brown, 2017). Furthermore, ADCs can engage the immune system through multiple mechanisms, including antibody-dependent cellular cytotoxicity (ADCC), antibody-dependent cellular phagocytosis (ADCP), and complement-dependent cytotoxicity (CDC) (Khera and Thurber, 2018; Liu et al., 2020) (Figure 1C). In addition, various chemotherapeutic agents have been shown to induce immunogenic cell death (ICD), which is characterized by the release of danger-associated molecular patterns (DAMPs) and subsequent reactivation of anti-tumor immune responses typically suppressed by the TME. This insight provides a strong rationale for investigating combination therapies that integrate ADCs with additional immune-stimulatory strategies (Gardai et al., 2015).

## 4 The application of approved ADC drugs in hematological tumors

The initial ADCs that entered the market were predominantly used for hematological tumors. Currently, seven ADCs have been approved for hematological tumors, providing a novel option for later-stage treatment.

### 4.1 Gemtuzumab ozogamicin (Mylotarg ®, Pfizer)

Chemotherapy and hematopoietic stem-cell transplantation (HSCT) are among the limited therapeutic options available for AML patients (Alibhai et al., 2009; Mayer et al., 1994; Fernandez et al., 2009; Pulte et al., 2009). More than 90% of AML patients exhibit leukemia cells with high CD33 expression levels, thereby rendering CD33 an attractive target for ADC drugs.

Gemtuzumab ozogamicin, as the first globally approved ADC, consists of a humanized IgG4 mAb targeting CD33 and linked to ozogamicin with a cleavable linker, AcBut (Appelbaum and Bernstein, 2017). The cytotoxic agent ozogamicin is a semi-synthetic derivative of calicheamicin, produced through microbial fermentation and subsequent chemical modification. In 2000, the FDA authorized gemtuzumab ozogamicin for treating CD33-positive R/R AML (Bross et al., 2001). However, due to linker instability, ozogamicin was prematurely released prior to reaching target cells, resulting in severe toxicity. Following its initial launch, multiple clinical studies identified significant adverse effects, leading to its withdrawal from the market in 2010 (Petersdorf et al., 2013; Ricart, 2011).

With the potential benefits of gemtuzumab ozogamicin in AML therapy, subsequent comprehensive clinical trials provided additional supporting data. The ALFA-0701 trial was a multi-center, randomized phase III study involving 271 newly diagnosed adult AML patients with CD33 expression (Lambert et al., 2019). Results demonstrated that the event-free survival (EFS) for the gemtuzumab ozogamicin combination group was 17.3 months compared to 9.5 months for the chemotherapy group (Lambert et al., 2019). The AML-19 trial was another multi-center, randomized, open-label phase III study comparing gemtuzumab ozogamicin monotherapy with best supportive care (BSC). The study revealed that the median overall survival (OS) for patients receiving gemtuzumab ozogamicin was 4.9 months *versus* 3.6 months for those receiving BSC (Amadori et al., 2016). Consequently, in 2017, the FDA re-approved gemtuzumab ozogamicin combination with chemotherapy for the treatment of newly diagnosed AML patients. This decision was motivated by the unmet clinical needs for AML treatment, given the limited options available for this condition. Currently, gemtuzumab ozogamicin remains the sole approved ADC for AML treatment.

### 4.2 Brentuximab vedotin (Adcetris®, Seagen)

Both HL and sALCL represent aggressive lymphomas that can potentially be cured with first-line multi-agent chemotherapy regimens (Stein et al., 2000). However, patients who experience refractory or relapsed (R/R) disease following initial treatment exhibit a poor prognosis. Given the high expression on HL and sALCL cells and its relatively low expression on normal cells, CD30 is considered an ideal target for anti-tumor therapy.

Brentuximab vedotin combines a chimeric IgG1 mAb targeting CD30, conjugated to MMAE via a protease-cleavable linker (Mc-Val-Cit-PABC), with a DAR of 4 (Katz et al., 2011; Francisco et al., 2003; Sanderson et al., 2005). Brentuximab vedotin remains stable in the bloodstream and, following its internalization by CD30-positive tumor cells, releases MMAE to disrupt tubulin polymerization and thereby inhibit cell division, ultimately leading to apoptosis (Francisco et al., 2003). Moreover, due to its inherent membrane permeability, MMAE can accumulate in the extracellular space and exhibit cytotoxic effects on neighboring CD30-negative bystander cells (Okeley et al., 2010). Additionally, brentuximab vedotin may potentially activate the initiation of anti-tumor immune response, thus achieving anti-tumor effects (Müller et al., 2014; Cao et al., 2017; Heiser et al., 2024).

The FDA first approved brentuximab vedotin for the treatment of R/R CD30-positive HL and sALCL in 2011 (Younes et al., 2012a; O'Reilly and Paulson, 2009). The phase II trial demonstrated the overall response rates (ORR) of brentuximab vedotin were 75% for HL and 86% for sALCL. In 2015, the phase III AETHERA trial established brentuximab vedotin as a consolidative treatment option for adult patients with classical HL at high risk of relapse or progression after autologous HSCT (Moskowitz et al., 2015). Patients treated with brentuximab vedotin showed a median progression-free survival (PFS) of 43 months, compared to 24 months in the control group (p = 0.001). Moreover, the randomized phase III ALCANZA trial provided significant evidence supporting the use of brentuximab vedotin in cutaneous T-cell lymphoma, demonstrating a PFS of 16.7 months *versus* 3.5 months in the single-agent chemotherapy arm (Prince et al., 2017). Compared with patients receiving chemotherapy alone, those undergoing brentuximab vedotin + chemotherapy demonstrated a 23% lower risk of disease progression, mortality, or the need for new treatment initiation (Straus et al., 2020). The ECHELON-3 phase III study showed the brentuximab vedotin group demonstrated a significantly longer median OS of 13.8 months compared to 8.5 months in the control group (p = 0.0085), longer median PFS (4.2 months vs. 2.6 months) and higher ORR (64.3% vs. 41.5%) (Bartlett et al., 2025). Therefore, the FDA approved brentuximab vedotin in combination with lenalidomide and rituximab for adult patients with R/R large B-cell lymphoma (LBCL) who are ineligible for HSCT or CAR T-cell therapy. With the expanding treatment indications, brentuximab vedotin is expected to play a critical role in oncology, offering new hope to patients.

### 4.3 Loncastuximab tesirine (Zynlonta®, ADC therapeutics)

CD19 is widely expressed throughout all stages of B-cell development and differentiation, from pre-B cells to plasma cells, with particularly high levels observed in malignant B cells, making it an ideal therapeutic target for B-cell malignancies (Wang et al., 2012; Jin et al., 2022; Maddocks, 2021). Table 3 outlines the therapeutic application of approved ADCs in B cell malignancies, categorized by their target antigens.

Loncastuximab tesirine consists of a humanized IgG1κ mAb targeting CD19 linked to SG3199 via a cleavable linker (Mal-PEG8-Val-Ala-PABC), with an average DAR of 2.3 (Zammarchi et al., 2018; Mullard, 2021). SG3199 is a cytotoxic PBD dimer alkylating agent. In April 2021, the FDA approved loncastuximab tesirine for treating adult patients with R/R LBL based on the LOTIS-2 study. This single-arm phase II trial indicated that among patients treated with loncastuximab tesirine, the ORR was 48.3%, with a CR rate of 24.1%. At a median follow-up of 7.3 months, the median duration of response (DOR) was 10.3 months. The most common grade 3 or higher treatment-related adverse effects (TRAEs) included neutropenia (26%), thrombocytopenia (18%), and elevated gamma-glutamyl transferase levels (17%) (Caimi et al., 2021). Recent studies investigating the combination of loncastuximab tesirine with chemotherapy for the treatment of R/R follicular lymphoma (FL) have also achieved promising results (Alderuccio et al., 2025). A confirmatory phase III clinical trial, ADCT-402–311, is currently underway to evaluate the efficacy and safety of loncastuximab tesirine in combination with rituximab *versus* rituximab plus gemcitabine and oxaliplatin for patients with R/R diffuse large B-cell lymphoma (DLBCL), which are expected to support loncastuximab tesirine combination therapy for second-line treatment of R/R DLBCL in the future.

### 4.4 Polatuzumab vedotin (Polivy®, Roche)

Globally, approximately 150,000 new cases of DLBCL are diagnosed annually, representing roughly 30% of all NHL (Sehn and Salles, 2021). While initial standard treatment (R-CHOP) achieves favorable outcomes in many patients, approximately 40% of patients experience relapse or resistance to therapy (Sehn and Salles, 2021; Yuen et al., 2024). CD79b, a key regulator of B-cell receptor expression and trafficking, is consistently expressed on the surface of nearly all B cells and is detectable in over 90% of NHL malignancies (Pfeifer et al., 2015; Wang et al., 2023), which makes CD79b a critical target for B-cell malignancies (Lenk et al., 2021; Burger and Wiestner, 2018; Polson et al., 2007).

Polatuzumab vedotin is an ADC composed of a mAb targeting CD79b linked to MMAE through a cleavable linker (Mc-Val-Cit-PABC), with an average DAR of 3.5 (Deeks, 2019; Fuh et al., 2017). Polatuzumab vedotin was first approved by the FDA in June 2019 for the treatment of adult patients with R/R DLBCL who had received at least two prior lines of therapy, when used in combination with bendamustine and rituximab (Pola-BR). This approval was based on the findings from an open-label, global, multicenter, phase Ib/II clinical trial (GO29365) (Deeks, 2019; Urquhart, 2019). After a median follow-up of 22.3 months, patients treated with Pola-BR demonstrated significantly improved outcomes compared to those receiving BR alone, with an ORR of 45.0% *versus* 17.5%, median PFS of 9.5 months *versus* 3.7 months, and median OS of 12.4 months *versus* 4.7 months (Sehn et al., 2020). In 2023, the approval of polatuzumab vedotin for first-line treatment of DLBCL in the US and China marked the first major advancement in 2 decades, which was primarily supported by the robust positive results from the phase III POLARIX study (Tilly et al., 2022). It aimed to evaluate the efficacy and safety of polatuzumab vedotin in combination with R-CHP compared to R-CHOP in previously untreated DLBCL patients. Data from the trial revealed that the 2-year PFS rates were 76.7% for Pola-R-CHP *versus* 70.2% for R-CHOP. Furthermore, the relative risk of disease progression, recurrence, or death was reduced by 27% with Pola-R-CHP.

Nonetheless, approximately 20%–40% of patients with LBCL continue to experience R/R disease despite treatment with Pola-R-CHP. Recently, a phase Ib/II clinical trial (NCT03671018) evaluated the efficacy and safety of Mosunetuzumab, a CD3xCD20 bispecific antibody, in combination with polatuzumab vedotin (mosun-pola) for treating R/R LBCL (Budde et al., 2024). The study demonstrated an ORR of 59.2%, a CR of 45.9%, a median PFS of 11.4 months, and a median OS of 23.3 months (Budde et al., 2024). Regarding safety, the adverse event profile of the combination therapy is consistent with those observed for the individual agents. This study provides preliminary evidence supporting the efficacy and safety of the mosun-pola regimen in LBCL, establishing a foundation for its potential clinical application. However, further research is necessary to investigate the resistance mechanisms linked to this regimen, optimize the treatment strategy and sequence, and conduct randomized controlled trials to comprehensively evaluate its clinical significance.

### 4.5 Belantamab mafodotin (Blenrep®, GSK)

Despite significant progress in the development of proteasome inhibitors, immunomodulatory drugs, and CD38-targeted antibodies, nearly all patients with multiple myeloma (MM) eventually relapse, with a 5-year OS of approximately 50% (Cowan et al., 2022). The need for innovative therapeutic strategies in this field remains unmet. B-cell maturation antigen (BCMA) is a transmembrane glycoprotein and a member of the TNFR superfamily. BCMA expression is primarily restricted to malignant plasma cells (Neri et al., 2024; Sellner et al., 2020).

Belantamab mafodotin comprises a humanized anti-BCMA mAb connected through a non-cleavable linker to MMAF, with an average DAR of 4 ^18^. In August 2020, the FDA approved belantamab mafodotin for treating R/R MM who had progressed after at least three lines of therapy based on the DREAMM-2 trial results (Sellner et al., 2020; Lonial et al., 2020). Results demonstrated that belantamab mafodotin monotherapy achieved an ORR of 35%. However, in the phase III DREAMM-3 trial, belantamab mafodotin monotherapy did not demonstrate superior efficacy compared to the control group, leading to its withdrawal from the market.

In May 2025, the combination therapy incorporating belantamab mafodotin was granted approval in Japan based on the positive results from two key phase III clinical trials: DREAMM-7 and DREAMM-8 (Lonial et al., 2020; Trudel and Stewart, 2024). The DREAMM-7 trial was designed to evaluate the efficacy and safety of belantamab mafodotin in combination with bortezomib and dexamethasone (BVd) *versus* daratumumab in combination with bortezomib and dexamethasone (DVd) in patients with R/R MM. The results demonstrated that the median PFS in the BVd group was significantly prolonged to 36.6 months, compared to 13.4 months in the DVd group. Furthermore, the 3-year OS rates were 74% and 60%, respectively. The DREAMM-8 trial assessed the efficacy and safety of belantamab mafodotin in combination with pomalidomide and dexamethasone (BPd) compared to bortezomib in combination with pomalidomide and dexamethasone (PVd). The median PFS in the BPd group had not yet been reached, whereas it was 12.7 months for the PVd group (p < 0.001). At a median follow-up of 21.8 months, 71% of patients in the BPd group remained alive and free of disease progression after 1 year, compared to 51% in the PVd group. Regarding safety and tolerability, the findings for the belantamab mafodotin combination therapy were largely consistent with the established profiles of the individual component drugs. The regimen demonstrates consistent efficacy even in the most challenging patient populations, including those resistant to lenalidomide, while maintaining an exemplary safety profile through optimized dosing protocols (Dimopoulos et al., 2023; McCurdy et al., 2024).

### 4.6 CD22-targeting ADC

CD22 is a key factor in the development, differentiation, and function of B cells, and is expressed in 60%–90% cases of B-ALL (Singh et al., 2021; Lanza et al., 2020; Shah et al., 2015) as well as in the majority of hairy cell leukemia (HCL) cells (Grever et al., 2014). CD22 exhibits rapid internalization upon binding to its cognate antigen, representing an attractive target for the development of ADCs. As of the latest updates, two CD22-ADCs have received global marketing approval.

#### 4.6.1 Inotuzumab ozogamicin (Besponsa®, Pfizer)

B-ALL arises from immature B lymphocyte precursor cells and is characterized by the clonal expansion of atypical lymphoblasts in the bone marrow. The standard first-line treatment for B-ALL is chemotherapy; however, this approach may result in drug resistance or significant adverse effects. Consequently, there is an urgent need for novel therapeutic agents or enhanced treatment strategies.

Inotuzumab ozogamicin is an ADC targeting CD22, which is covalently linked to calicheamicin via AcBut, and the process results in an average DAR of 5–7 (Short et al., 2024). The linker resembles that of gemtuzumab ozogamicin, which features a pH-sensitive design based on hydrazone and disulfide bonds. However, the current linker enhances stability by introducing increased steric hindrance near the disulfide bond, thereby mitigating safety concerns associated with premature payload release. Inotuzumab ozogamicin was approved by the FDA in 2017 for treating adults with R/R B-ALL based on the results of a phase III INO-VATE ALL trial, which enrolled 326 participants who were randomized to receive either inotuzumab ozogamicin or conventional chemotherapy. The study demonstrated that inotuzumab ozogamicin significantly improved outcomes compared to chemotherapy, with a median PFS of 5.0 months *versus* 1.8 months, a median OS of 7.7 months *versus* 6.7 months, and a CR of 81% *versus* 29% (Kantarjian et al., 2019). Furthermore, the phase II INITIAL-1 trial provides strong evidence supporting inotuzumab ozogamicin as an initial treatment option for older patients with B-ALL. All 45 patients achieved CR, with 53% and 71% showing no measurable residual disease after the second and third inductions, respectively. After a median follow-up of 2.7 years, the EFS at one and 3 years were 88% and 55%, while OS were 91% and 73%, respectively. Common TRAEs included hematological abnormalities and elevated liver enzymes (Stelljes et al., 2024). These findings provide a rationale for incorporating inotuzumab ozogamicin into first-line regimens for older patients with B-ALL.

#### 4.6.2 Moxetumomab pasudotox (Lumoxiti®, AstraZeneca)

HCL represents a rare and indolent tumor affecting mature B lymphocytes, characterized by hair-like protrusions on the cell surface (Falini and Tiacci, 2024). While standard regimen exhibit a favorable prognosis for HCL treatment, long-term follow-up data reveal that approximately 30%–40% of patients eventually relapse. For R/R HCL patients, no alternative therapeutic options were available for over 2 decades.

**FIGURE 4:**
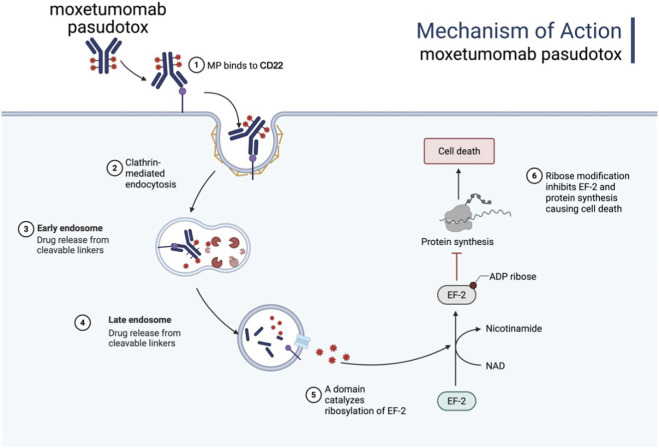
The mechanism of action of Moxetumomab pasudotox. It is internalized into the cell via endocytosis, forming an endosome. Within the acidic environment of the endosome, the immunotoxin releases the PE38 toxin, which inhibits protein synthesis by targeting EF-2, ultimately inducing apoptosis or necrosis in tumor cells. Figure was created by Biorender.com.

Moxetumomab pasudotox is composed of the variable fragment (Fv) of a recombinant anti-CD22 mAb, which is genetically fused to the 38 kDa cytotoxic domain of *pseudomonas* exotoxin A (PE38) via a cleavable linker (Mc-Val-Cit-PABC). Upon cellular internalization, PE38 catalyzes the ADP-ribosylation of the dipeptide residue in elongation factor-2, thereby disrupting protein synthesis and inducing apoptosis, leading to the eradication of tumor cells, as shown in Figure 4 (Kreitman and Pastan, 2011). Moxetumomab pasudotox was approved by FDA for the treatment of adult patients with R/R HCL in September 2018. In a phase III trial (Study 1053), 80 patients with R/R HCL who had received at least two prior therapies were administered moxetumomab pasudotox (Kreitman et al., 2018). Following a median follow-up of 16.7 months, 41% achieved CR, 34% achieved PR, and the ORR was 75%, including 80% of patients achieving hematologic response. Overall, moxetumomab pasudotox exhibited a highly durable response in R/R HCL patients after multiple lines of treatment, with an acceptable safety profile [81].

## 5 The application of approved ADC drugs in solid tumors

With advancements in technology, ADCs have overcome their application limitations and are now equally effective in treating both solid and hematological tumors.

### 5.1 HER-2-targeting ADC

Human epidermal growth factor receptor-2 (HER2) shows significant overexpression in approximately 15%–20% of breast cancer (BC) patients (Hamilton et al., 2021). To date, the THP regimen (trastuzumab + pertuzumab + chemotherapy) has served as the standard first-line treatment for HER2-positive metastatic BC for over a decade. Although the pathologic CR rate associated with the THP regimen is approximately 50% (Gianni et al., 2012; Gao et al., 2025; Shao et al., 2020), 22%–25% of patients with HER2-positive metastatic BC exhibit either primary or secondary resistance to HER2-targeted therapies (Lux et al., 2018; Choong et al., 2020). However, taxane-based chemotherapy frequently causes severe side effects, such as neurotoxicity and bone marrow suppression, which lead to treatment interruptions or dose reductions, ultimately affecting therapeutic outcomes. Therefore, identifying alternative regimens that maintain efficacy while reducing toxicity and improving patient prognosis remains a critical priority. HER2 is one of the most competitive targets in the ADC field, with indications spanning major cancer types such as lung cancer, gastric cancer, and breast cancer. To date, four HER2-targeted ADCs have been approved globally.

#### 5.1.1 Trastuzumab emtansine (Kadcyla®, Roche)

Trastuzumab emtansine (T-DM1) is composed of the humanized HER2-mAb trastuzumab and DM1 conjugated via a non-cleavable linker, with an average DAR of 3.5 (Yao et al., 2015; Ogitani et al., 2016; Junttila et al., 2011). As the world’s first ADC targeting HER2, T-DM1 received FDA approval in February 2013 for use in HER2-positive locally advanced or metastatic (la/m) BC patients who had previously received trastuzumab and taxane-based therapy (Ballantyne and Dhillon, 2013; Chen et al., 2023; Amiri-Kordestani et al., 2014). Results of the phase III EMILIA trial (NCT00829166) demonstrated that T-DM1 significantly prolonged median PFS to 9.6 months compared to 6.4 months in the lapatinib + capecitabine group, reducing the risk of disease progression or death by 35%. Additionally, T-DM1 provided a significant OS benefit (30.9 months vs. 25.1 months). The ORR was also higher in the T-DM1 group (43.6% vs. 30.8%) (Verma et al., 2012; Diéras et al., 2017). The KATHERINE phase III study (NCT01772472) revealed significantly prolonged invasive disease-free survival with adjuvant T-DM1 compared to trastuzumab for HER2-positive BC patients with residual invasive disease following neoadjuvant trastuzumab and taxane-based therapy (von Minckwitz et al., 2019). Based on these findings, T-DM1 gained additional approval for use in patients with HER2-positive BC who showed residual invasive disease after neoadjuvant therapy in May 2019.

#### 5.1.2 Trastuzumab deruxtecan (Enhertu®, Daiichi Sankyo)

Trastuzumab deruxtecan (T-DXd) consists of trastuzumab and delivers a highly potent Top 1 inhibitor (DXd) as its payload (Cortés et al., 2022), with an average DAR of eight and a cleavable linker (GGFG). T-DXd exhibits robust anti-tumor activity and demonstrates a bystander effect, enabling the elimination of neighboring tumor cells (Angelastro et al., 2022). Owing to its exceptional product design and remarkable clinical performance, T-DXd is anticipated to replace T-DM1 as the new ADC king.

From a later-line option to first-line treatment, each advancement of T-DXd has significantly transformed the landscape of HER2-positive BC. In the phase II DESTINY-Breast01 trial, T-DXd monotherapy demonstrated promising efficacy, showing an ORR of 61.4%, a median PFS of 19.4 months, and an 18-month OS of 74% (Tamura et al., 2019; Modi et al., 2020). Therefore, the FDA approved T-DXd for later-line treatment in HER2-positive metastatic BC in 2019 (Hatschek et al., 2021; Keam, 2020). In the phase III DESTINY-Breast03 trial (NCT03529110), which compared T-DXd with T-DM1 in HER2-positive metastatic BC patients previously treated with one anti-HER2 regimen, T-DXd demonstrated superior efficacy with a median PFS of 29.0 months *versus* 7.2 months, a 36-month PFS rate of 45.7% *versus* 12.4%, and a median OS of 52.6 months *versus* 42.7 months, respectively (Cortés et al., 2022). TRAEs were consistent with previous analyses. Consequently, T-DXd has been approved in over 75 countries worldwide for second-line treatment in HER2-positive BC patients. Recently, the phase III DESTINY-Breast09 trial demonstrated groundbreaking results when T-DXd was combined with pertuzumab (T-DXd + P), potentially reshaping the first-line treatment paradigm for BC.

The trial demonstrated that, compared to the current first-line standard therapy (THP), T-DXd + P significantly achieved greater efficacy in HER2-positive BC patients, with a median PFS of 40.7 months *versus* 26.9 months (P < 0.00001), a 24-month PFS rate of 70.1% *versus* 52.1%, and a median DOR of 39.2 months *versus* 26.4 months (Tolaney et al., 2025a). Furthermore, T-DXd has achieved remarkable success across multiple cancer types. DESTINY-PanTumor02, as the international multi-center investigation across seven solid tumor types, demonstrated median DOR of T-DXd reaching 22.1 months, equating to nearly 2 years of sustained effectiveness (Meric-Bernstam et al., 2024). The HERALD phase II trial was the first to confirm the broad-spectrum efficacy of T-DXd in 12 advanced HER2-positive solid tumors detected by plasma circulating tumor DNA (ctDNA). Over half of the patients experienced significant tumor shrinkage, achieving an ORR of 56.5% and a CR of 14.5% (Yagisawa et al., 2024). These results surpass traditional chemotherapy (typically <15%) and even outperform certain targeted therapies for specific cancers. However, given the inherent limitations of ctDNA analysis—including reduced sensitivity and its capacity to reflect only the systemic tumor genomic profile—discrepancies between ctDNA and the diagnostic gold standard (tissue-based confirmation) could introduce HER2 status biases. In colorectal cancer specifically, the DESTINY-CRC02 study revealed the superior performance of T-DXd with a 37.8% ORR—significantly exceeding conventional targeted agents (typically <20% ORR)—accompanied by an 11.3-month median DOR that addresses critical unmet needs in treatment-resistant populations (Raghav et al., 2024). DESTINY-Lung01 set a new benchmark in lung cancer treatment. In Non-Small Cell Lung Cancer (NSCLC), the ORR of T-DXd for HER2-mutant patients was 55%, with a median DOR of 9.3 months, marking a new era in targeted therapy. Notably, T-DXd overcomes traditional therapeutic limitations through enhanced blood-brain barrier penetration, achieving a 42.9% intracranial response rate in patients with brain metastases (Smit et al., 2024).

Moreover, T-DXd is the only approved therapy for treating patients with HER2-low and HER2-ultralow expressing BC. HR-positive, HER2-negative BC represents the most common subtype, accounting for approximately 70% of all BCs. Although classified as HER2-negative, many patients exhibit some degree of HER2 expression. Positive results from the phase III DESTINY-Breast06 study assessed the efficacy and safety of T-DXd *versus* standard chemotherapy (capecitabine, paclitaxel, and nab-paclitaxel) in HR-positive, HER2-low or HER2-ultralow expressing metastatic BC patients who progressed after endocrine therapy. Subgroup analysis of the HER2-low population showed that the median PFS in the T-DXd group was significantly longer (13.2 vs. 8.1 months, p < 0.0001), with a higher proportion of patients reaching 12 months of OS (87.0% vs. 81.1%) and a higher confirmed ORR (57.3% vs. 31.2%) (Bardia et al., 2024a). Based on this, T-DXd was approved by the FDA as a single-agent therapy for HR-positive, HER2-low expressing or HER2-ultralow expressing BC patients in January 2025.

#### 5.1.3 Trastuzumab rezetecan (艾维达®, Hengrui)

Trastuzumab rezetecan consists of a trastuzumab conjugated to Top I inhibitors (SHR169265) via a cleavable linker (MC-Gly-Gly-Phe-Gly). Compared with DXd, SHR169265 exhibits superior membrane permeability and cytotoxicity with a reduced DAR of 6. Notably, trastuzumab rezetecan features an innovative chiral cyclopropyl structure between the linker and the toxin payload, thereby enhancing chemical stability and reducing side effects associated with premature toxin release (Zhang et al., 2023; Li Z. et al., 2024).

In May 2025, the National Medical Products Administration (NMPA) approved trastuzumab rezetecan as a later-line treatment option for adult patients with unresectable, la/m HER2-mutant NSCLC who had demonstrated resistance to platinum-based chemotherapy and immunotherapy. In the phase II trial (NCT04818333) involving 94 patients with locally advanced or metastatic (la/m) HER2-mutant NSCLC treated with trastuzumab deruxtecan, an ORR of 74.5% and a DCR of 98.9% were observed, along with a median DOR of 9.8 months and median PFS of 11.5 months 128, while common TRAEs primarily included hematologic abnormalities, which were largely manageable through dose modifications or symptomatic interventions (Li Z. et al., 2025). In addition to its recent approval for NSCLC, trastuzumab rezetecan has also been approved for multiple phase III clinical trials targeting other tumors, including BC, gastric cancer, and colorectal cancer (Yao et al., 2024). As a new ADC drug, trastuzumab rezetecan has shown encouraging safety and effectiveness in different types of HER2-mutant advanced solid tumors, possibly providing a novel therapeutic alternative for a wider range of tumor patients.

#### 5.1.4 Disitamab vedotin (Aidixi®, RemeGen)

Disitamab vedotin comprises a mAb distinct from trastuzumab, a cleavable linker (Mc-Val-Cit), and a cytotoxic payload MMAE, with an average DAR of approximately 4 (Yaghoubi et al., 2021; Zhu et al., 2021; Shi et al., 2022).

As the first developed ADC in China, disitamab vedotin has demonstrated unparalleled clinical value through its unique positioning and innovative advancements in a highly competitive landscape. China is the country with the largest patient population with gastric cancer/gastroesophageal junction adenocarcinoma (GC/GEJC), accounting for 42.6% of the worldwide incidence (Chen Y. et al., 2025). HER2 plays a crucial role in the prognosis and survival of GC/GEJC (Li et al., 2022). In June 2021, disitamab vedotin received regulatory approval from NMPA for the treatment of patients with la/m GC/GEJC who had previously undergone at least two systemic chemotherapy regimens. The approval was based on the results of a single-arm study (NCT03556345) involving 127 eligible GC/GEJC patients, which demonstrated an ORR of 24.4%, a median DOR of 4.7 months, a median PFS of 4.1 months, and an OS of 7.9 months (Peng et al., 2021). Furthermore, according to the single-arm RC48-C005 study, the FDA approved disitamab vedotin for treating HER2-positive la/m urothelial carcinoma (UC) who had progressed after at least one prior systemic chemotherapy regimen. The RC48-C005 study showed the ORR of disitamab vedotin was 51.2%, the DOR was 6.9 months, the median PFS was 6.9 months, and the OS was 13.9 months (Sheng et al., 2021). At the 2021 ASCO conference, data from the phase Ib/II (RC48-C014) trial showed that the combination of disitamab vedotin and immunotherapy for HER2-expressing UC achieved an impressive ORR of 94.1%. The 3-year follow-up revealed a median OS of 33.1 months, which exceeded the previous first-line platinum-based chemotherapy OS of approximately 13 months by 20 months (Zhou et al., 2025). These figures represent the highest ORR and longest OS reported to date in the first-line treatment of advanced UC.

Despite the dominance of T-DM1 and the exceptional performance of T-DXd in BC therapy, disitamab vedotin has successfully established itself in a distinct niche. The liver is the third most frequent site of BC metastasis, impacting approximately 45% of HER2-positive advanced BC patients (Rashid et al., 2021). Due to various constraints, this subfield remains challenging, with limited effective treatment options and significant unmet clinical needs. In May 2025, the NMPA approved a new indication for disitamab vedotin for treating HER2-positive advanced BC with liver metastasis. This approval was supported by a phase III trial (RC48-C006, NCT03500380) (Qu et al., 2024). In which disitamab vedotin significantly reduced the risk of disease progression or death by 44%, with a median PFS of 9.9 months *versus* 4.9 months. Regarding OS, although the data are not yet mature, a clear trend of benefit has been observed in the disitamab vedotin group, with median OS not yet reached *versus* 25.92 months in the control group (Qu et al., 2024). Disitamab vedotin is the first approved HER2-ADC to achieve positive results in a confirmatory phase III study for HER2-positive advanced BC with liver metastasis.

### 5.2 Trop-2-targeting ADC

Trophoblast cell-surface antigen-2 (Trop-2) plays a critical role in Ca^2+^ signaling in tumor cells, exhibiting high expression levels across various tumor tissues and demonstrating robust internalization activity, making it an emerging and promising molecular target for targeted therapy. For example, up to 80%–90% of triple-negative breast cancer (TNBC) cells exhibit high levels of Trop-2 expression (Li et al., 2021). TROP-2 is also highly expressed in lung cancer cells and actively participates in multiple signaling pathways involved in tumor progression. Table 2 summarized the comparison of therapeutic efficacy across different ADCs targeting CD22, HER2, and TROP-2.

**TABLE 2:** Comparison of ADC drugs targeting the same antigens in a specific tumor type.

Target	Tumor	Name	Trial design	Indication	Outcomes
CD22	B-cell malignant tumors	Inotuzumab ozogamicin	phase III	R/R ALL	mOS is 7.7 months, mPFS is 5.0 months (Kantarjian et al., 2019)
		Moxetumomab pasudotox	phase III	adult patients with R/R HCL	ORR was 75% (Kreitman et al., 2018)
HER2	NSCLC	Trastuzumab rezetecan	phase II	adult patients with la/m HER2-mutant NSCLC	ORR is 74.5%, DCR reached 98.9%, mDOR is 9.8 months, mPFS is 11.5 months (Li et al., 2025c)
		Trastuzumab deruxtecan	phase II	patients with HER2-overexpressing or HER2-mut u/mNSCLC	ORR is 34.1%, mOS is 11.2 months, mPFS is 6.7 months, mDOR is 6.2 months (Smit et al., 2024)
	Breast cancer	Trastuzumab emtansine	phase III	HER2^+^ mBC patients who had received one prior anti-HER2 regimen	The mPFS was 29.0 vs. 7.2 months, the 36-month PFS rate was 45.7% vs. 12.4%, and the mOS was 52.6 vs. 42.7 months for T-DXd vs. T-DM1 (Cortés et al., 2022)
		Trastuzumab deruxtecan			
TROP-2	NSCLC	Sacituzumab tirumotecan	phase I/II	R/R la/m NSCLC	ORR is 44%, the median DOR is 9.3 months, 6-month DOR is 77% (Fang et al., 2023)
		Datopotamab deruxtecan	phase III	la/m NSCLC	non-squamous subgroup: mPFS is 5.6 months, ORR is 31.2% squamous subgroup: mPFS is 4.4 months, ORR is 12.8% (Ahn et al., 2025)
	Breast cancer	Datopotamab deruxtecan	phase III	HR^+^/HER2^–^la/mBC who have previously been treated with endocrine therapy and chemotherapy	ORR is 36%, mPFS is 13.2 months, mDOR is 6.7 months (Bardia et al., 2025a; Bardia et al., 2024c; Bardia et al., 2025b)
	TNBC	Sacituzumab govitecan	phase III	adult patients with la/m TNBC who have undergone at least two prior systemic treatments	mPFS is months, mOS was 11.8 months (Bardia et al., 2021c)
		Sacituzumab tirumotecan		adult patients with la/m TNBC who have received at least two prior systemic therapies	ORR is 45.4%, mPFS is 5.7 months, DOR is 7.1 months, The 12-month OS rates were 57.8% (Xu et al., 2024)

#### 5.2.1 Sacituzumab govitecan (Trodelvy®, immnomedics)

Sacituzumab govitecan is an ADC comprising a humanized IgG1 mAb targeting Trop-2, linked to the Top1 inhibitor SN-38 via a pH-sensitive linker (CL2A) (Angelastro et al., 2022). Several key clinical trials have highlighted the exceptional effectiveness of sacituzumab govitecan in managing advanced TNBC. Notably, the phase III ASCENT trial indicated that sacituzumab govitecan decreased the risk of disease progression or death by 59% compared to conventional chemotherapy in TNBC. Specifically, sacituzumab govitecan improved median PFS from 1.7 months to 4.8 months and median OS from 6.9 months to 11.8 months over conventional chemotherapy (Rugo et al., 2023; Bardia et al., 2021a). Based on these results, sacituzumab govitecan received FDA approval in 2022 for treating adult patients with unresectable, la/m TNBC who have undergone at least two prior systemic treatments (Syed, 2020). The phase IIb EVER-132-001 trial further validated the efficacy and safety of sacituzumab govitecan in Chinese TNBC patients who had failed more than two prior chemotherapy regimens. Results showed that sacituzumab govitecan monotherapy achieved an ORR of 33.3%, with a median DOR lasting 7.7 months (Xu et al., 2023). Consequently, sacituzumab govitecan has been widely recommended by various domestic and international guidelines, establishing the standard of care for second-line and subsequent treatments of TNBC. As research has advanced, the applications of sacituzumab govitecan have expanded beyond TNBC to other solid tumors with high Trop-2 expression, including NSCLC, UC, and endometrial cancer (Powles et al., 2025; Bardia et al., 2021b; Paz-Ares et al., 2024; Santin et al., 2024).

In the first-line treatment of la/m TNBC, the preferred therapeutic approach involves chemotherapy combined with PD-L1 inhibitors (Tolaney et al., 2025b; Mittendorf et al., 2020). At the 2025 ASCO meeting, the randomized, phase III ASCENT-04/KEYNOTE-D19 trial demonstrated that, in patients with PD-L1-positive advanced TNBC (Tolaney et al., 2025b), the combination of sacituzumab govitecan and pembrolizumab significantly improved PFS compared to chemotherapy plus pembrolizumab. This regimen reduced the risk of disease progression or death by 35%, with a median DOR of 16.5 months *versus* 9.2 months in the chemotherapy group, nearly doubling the response duration. These findings represents a paradigm shift toward the combination of ADCs and immunotherapy as a first-line strategy of TNBC, potentially setting a precedent for cross-tumor therapeutic approaches in the future.

#### 5.2.2 Sacituzumab tirumotecan (佳泰莱®, Sichuan Kelun)

Sacituzumab tirumotecan incorporates an anti-Trop-2 humanized IgG1 mAb, similar to that used in sacituzumab govitecan, and is conjugated via CL2A to a novel Top1 inhibitor, KL610023 (a derivative of belotecan). The cytotoxic potency of KL610023 is approximately 1.5 times greater than that of SN-38, with an average DAR of approximately 7.4 (Yin et al., 2025). Moreover, KL610023 can be released via enzymatic or acid-mediated cleavage, enabling dual activation through intracellular enzymatic hydrolysis and acidic TME (Li N. et al., 2025; Yin et al., 2025). Compared with sacituzumab govitecan, sacituzumab tirumotecan demonstrates superior performance in terms of effective payload exposure, half-life, and tumor control under the same conditions (Cheng et al., 2022).

In the phase III OptiTROP-Breast01 study (NCT05347134), sacituzumab tirumotecan was compared with investigator-selected chemotherapy in patients with la/m TNBC who had received two or more prior therapies (Yin et al., 2025). The median PFS was significantly longer in the sacituzumab tirumotecan group compared to the chemotherapy group (5.7 months vs. 2.3 months, p < 0.00001). The risk of disease progression or death was significantly reduced by 69% in the sacituzumab tirumotecan group. The 12-month OS rates were 57.8% and 35.2%, respectively. And the ORR in the sacituzumab tirumotecan group and chemotherapy group was 45.4% and 12.0%, respectively, while the DOR was 7.1 months and 3.0 months, respectively (Xu et al., 2024). Based on this study, the NMPA approved sacituzumab tirumotecan for the treatment of adult patients with unresectable, la/m TNBC who have received at least two prior systemic therapies (Xu et al., 2024).

The KL264-01 trial (NCT04152499) demonstrated the promising efficacy of sacituzumab tirumotecan monotherapy in patients with R/R la/m NSCLC (Ye et al., 2025; Fang et al., 2023). After a median follow-up of 11.5 months, the ORR was 44%, the median DOR was 9.3 months, 6-month DOR rate was 77%. For the subgroup with TKI-resistant EGFR-mut NSCLC, the ORR was 60%, DCR was 100%, median PFS was 11.1 months, and 9-month PFS was 66.7%. A total of 67.4% of patients had grade 3 or higher TRAEs. Subsequent phase III studies in patients with advanced NSCLC are currently being planned (Fang et al., 2023).

In addition, sacituzumab tirumotecan has demonstrated promising activity across various tumor types and is currently under clinical investigation, including endometrial cancer, platinum-resistant ovarian cancer (PROC), cervical cancer, gastroesophageal adenocarcinoma. The ongoing studies reflect the potential of sacituzumab tirumotecan in expanding treatment options for patients with difficult-to-treat cancers.

#### 5.2.3 Datopotamab deruxtecan (Datroway®, Daiichi Sankyo/AstraZeneca)

Datopotamab deruxtecan (Dato-DXd) is an ADC comprising another humanized anti-TROP-2 IgG1 mAb linked via a cleavable linker (GGFG) to DXd (Okajima et al., 2021; Bardia et al., 2024b). The linker in Dato-DXd releases the drug through specific enzymatic cleavage, ensuring high stability and enabling precise targeting of TROP2-positive tumor cells. Dato-DXd optimizes the DAR to 4, achieving a balance between efficacy and toxicity while maximizing the therapeutic window (Okajima et al., 2021).

The phase III, open-label, randomized TROPION-Breast01 study demonstrated that Dato-DXd significantly improved PFS and exhibited a favorable safety profile compared to investigator-selected chemotherapy in patients with hormone receptor-positive/HER2-negative (HR^+^/HER2^–^) BC who had progressed on endocrine therapy and chemotherapy. The confirmed ORR was 36% and 23%, and the median DOR was 6.7 months and 5.7 months in the Dato-DXd and chemotherapy groups, respectively. Notably, the incidence of grade 3 or higher TRAEs was lower with Dato-DXd than chemotherapy (20.8% vs. 44.7%) (Bardia et al., 2025a; Bardia et al., 2024c; Bardia et al., 2025b). Based on these results, Dato-DXd received its initial approval from the Pharmaceuticals and Medical Devices Agency (PMDA) in Japan in December 2024 and was subsequently approved by the FDA in January 2025.

Additionally, Dato-DXd has extended its clinical applications beyond BC to other solid tumors. In NSCLC, the phase III TROPION-Lung01 trial (NCT04656652) demonstrated that Dato-DXd significantly improved PFS compared to docetaxel in previously treated NSCLC patients (4.4 months vs. 3.7 months, respectively). Notably, the non-squamous subgroup achieved a median PFS of 5.5 months and a median OS of 14.6 months. Dato-DXd exhibited superior safety, with only 3% of patients experiencing grade 3 TRAEs, compared to 42% in the docetaxel arm (Ahn et al., 2025). Based on these findings, the FDA accepted the application of Dato-DXd for NSCLC in January 2025. Furthermore, the phase II TROPION-Lung05 trial (NCT0448414) reported an ORR of 42.7% and an intracranial DOR of 72% in EGFR-mutated NSCLC patients resistant to EGFR tyrosine kinase inhibitors (EGFR-TKIs) and platinum-based chemotherapy (Sands et al., 2025), leading to the FDA granting it breakthrough therapy designation. Furthermore, the phase I TROPION-PanTumor01 trial (NCT03401385) indicated an ORR of 25% in previously treated UC patients, with a median PFS of 6.9 months (Meric-Bernstam et al., 2025). Regarding safety, the incidence of interstitial lung disease was 3%, which can be effectively managed through dose adjustment and regular monitoring. Consequently, Dato-DXd is anticipated to become a cornerstone therapeutic option for multiple cancer types in the future.

### 5.3 Enfortumab vedotin (Padcev®, Seagen)

Patients with la/m UC who were ineligible for surgical intervention predominantly relied on chemotherapy as the standard treatment. Since 2016, the introduction of PD-(L)1 inhibitors has ushered in a new era of immunotherapy for UC management. Despite this advancement, therapeutic options remained limited for patients experiencing disease progression following PD-(L)1 inhibitor and platinum-based chemotherapy regimens (Nelson et al., 2021; Nadal et al., 2024). Nectin cell adhesion molecule-4 (Nectin-4), a member of the nectin family of cell adhesion molecules, exhibits high expression levels in UC and is strongly associated with unfavorable prognosis (Liu et al., 2021). As a consequence, Nectin-4 has emerged as a promising target for systemic therapy in UC (Challita-Eid et al., 2016). Table 3 outlines the therapeutic application of approved ADCs in UC, categorized by their target antigens.

**TABLE 3:** Clinical endpoints of ADCs.

Cancer	Targets	Name	Trial design	Indication	Clinical outcome
B-cell malignant tumors	CD30	Brentuximab vedotin	phase II	R/R la/m cHLand sALCL	cHL: ORR is 73%, CR is 34% (Younes et al., 2012b) sALCL: ORR is 86%, CR is 57% (Pro et al., 2012)
	CD19	Loncastuximab tesirine	phase II	adult patients with R/R DLBCL or high-grade B-cell lymphoma	ORR of is 48.3%, mDOR is 10.3 months (Caimi et al., 2021)
urothelial carcinoma	HER2	Disitamab vedotin	phase II	HER2-positive la/m UC	ORR is 51.2%, mPFS is 6.9 months, DOR is 6.9 months, OS is 13.9 months (Sheng et al., 2021)
	TROP-2	Datopotamab deruxtecan	phase I	previously treated UC patients	ORR is 25%, mPFS is 6.9 months (Meric-Bernstam et al., 2025)
	Nectin-4	Enfortumab vedotin	phase III	la/mUC patients who have failed platinum-based chemotherapy and PD-1/PD-L1 inhibitors	ORR is 41.32%, mOS is 12.9 months (Powles et al., 2021)

Enfortumab vedotin combines a human antibody against Nectin-4 with the cytotoxic MMAE through a cleavable linker (Mc-Val-Cit-PABC). The average DAR is roughly 3.8 (Bardia et al., 2025a; Challita-Eid et al., 2016). Enfortumab vedotin is the first FDA-approved ADC targeting Nectin-4 and represents the initial ADC approved specifically for UC. In 2019, the FDA granted approval for enfortumab vedotin based on data from the EV-201 trial for the treatment of adults with la/m UC who failed with a PD-(L)1 inhibitor and platinum-based chemotherapy. The single-arm phase II trial demonstrated an ORR of 44% and a DOR of 7.6 months following enfortumab vedotin treatment (Chang et al., 2021; Rosenberg et al., 2019). Prior to its approval, subsequent treatment options for these patients were severely constrained, with ORRs from conventional chemotherapy typically below 20%. The further phase III EV-301 study verified that enfortumab vedotin exhibited a significant survival advantage, with a median OS of 12.91 months *versus* 8.94 months in the chemotherapy group in la/m UC patients.

The EV-302 study evaluated the efficacy of enfortumab vedotin + pembrolizumab *versus* platinum-based chemotherapy in untreated patients with la/m UC. The combination of enfortumab vedotin + pembrolizumab extended the median PFS from 6.3 months to 12.5 months, nearly doubled the OS from 16.1 months to 31.5 months, and reduced the risk of death by 53% (Brave et al., 2024; Gupta et al., 2025). The clinical response achieved a historical peak, with an ORR of 68% in the combination group compared to 44% in the control group. Safety profiles between the two groups were comparable. Based on the positive outcomes of the EV-302 study, enfortumab vedotin in combination with pembrolizumab was approved by FDA in December 2023 and received endorsement from major international guidelines, including National Comprehensive Cancer Network (NCCN) and European Society for Medical Oncology (ESMO), as a first-line treatment option for la/m UC. The combination of enfortumab vedotin and pembrolizumab has replaced platinum-based chemotherapy as the new treatment paradigm, formally ushering in a new era in the chemotherapy-free treatment for la/m UC.

### 5.4 Tisotumab vedotin (Tivdak®, Genmab/Seagen)

The management of recurrent and metastatic cervical cancer remains a significant clinical challenge. The scarcity of effective standard therapeutic options introduces uncertainty regarding patient outcomes. Tissue factor (TF) plays a pivotal role in promoting tumor growth, angiogenesis, and accelerating metastasis. Studies have shown that TF is highly expressed in cervical cancer tissues, while demonstrates minimal or no expression in adjacent normal tissues, thereby establishing TF as a promising therapeutic targets (Zhao et al., 2018).

Tisotumab vedotin incorporates a fully human IgG1-κ mAb targeting TF, connected to the cytotoxin MMAE via a cleavable linker (Mc-Val-Cit-PABC), with a DAR of 4 (Markham, 2021; De, 2022; de Goeij et al., 2015). Besides the cytotoxic function of MMAE, tisotumab vedotin demonstrates ADCP and ADCC activities *in vitro*. In 2024, the FDA approved tisotumab vedotin for treating adult patients with recurrent or metastatic cervical cancer, which was based on data from the phase III InnovaTV 301 trial (NCT04697628) (Markham, 2021; Coleman et al., 2021). Compared to conventional chemotherapy, tisotumab vedotin demonstrated superior efficacy with a median OS of 11.5 *versus* 9.5 months, a median PFS of 4.2 *versus* 2.9 months, and a confirmed ORR of 17.8% *versus* 5.2%, while maintaining a comparable safety profile with Grade ≥3 TRAEs occurring in 52.0% *versus* 62.3% of patients (Vergote et al., 2024). In March 2025, tisotumab vedotin was officially approved in Japan for the treatment of advanced or recurrent cervical cancer that progresses after chemotherapy, which marks the first ADC specifically developed for cervical cancer and the first ADC targeting TF.

Ongoing studies are exploring the potential use of tisotumab vedotin for other solid tumors, including head and neck cancers (de Bono et al., 2019; Bakema et al., 2024), as well as its combination with other chemotherapeutic agents for managing recurrent or metastatic cervical cancer (Vergote et al., 2023).

### 5.5 Telisotuzumab vedotin (Emrelis®, AbbVie)

NSCLC continues to be the primary cause of cancer-related mortality worldwide (Rebecca et al., 2025; Gesthalter et al., 2022). c-MET is a receptor tyrosine kinase (RTK) that is frequently overexpressed in various solid tumors, including NSCLC. c-MET is encoded by the MET proto-oncogene and serves as the cellular receptor for hepatocyte growth factor (HGF) (Organ and Tsao, 2011). Abnormal activation of the c-MET/HGF signaling pathway leads to tumor progression, angiogenesis, invasive growth, metastasis, and resistance to treatment (Tong et al., 2016; Ma et al., 2003; Lennerz et al., 2011). Approximately 25% of patients with advanced NSCLC harbor c-Met overexpression, which is strongly associated with an unfavorable prognosis (Motwani et al., 2021; Wang et al., 2017). Therapeutic options have been limited for patients with non-squamous NSCLC who exhibit high c-MET protein overexpression and have undergone prior systemic therapy.

Telisotuzumab vedotin is a conjugate that combines an anti-c-Met mAb (ABT-700) with the cytotoxin MMAE, connected via a cleavable linker (Mc-Val-Cit-PABC), with the DAR of 3 (Fujiwara et al., 2021). In May 2025, the FDA approved telisotuzumab vedotin for adult patients with la/m non-squamous NSCLC characterized by c-MET overexpression. The phase II LUMINOSITY trial (NCT03539536), evaluating telisotuzumab vedotin in previously treated NSCLC patients with c-MET overexpression, demonstrated an ORR of 35% and a median DOR of 7.2 months 195, with a generally manageable and well-tolerated safety profile (Camidge et al., 2024). Further validation of clinical endpoints, including OS, is required in phase III trials. A global confirmatory phase III TeliMET NSCLC-01 trial (NCT04928846) for telisotuzumab vedotin in previously treated c-MET overexpressing NSCLC patients is currently ongoing.

### 5.6 Mirvetuximab soravtansine (Elahere®, AbbVie)

The development of resistance to platinum-based chemotherapy remains a significant clinical challenge in ovarian cancer (OC) (Indini et al., 2021; Alvarez Secord et al., 2025). OC that exhibits resistance to platinum-based therapies is classified as PROC, representing approximately one-third of all OC cases globally. Folate receptor alpha (FRα) facilitates the cellular uptake of folate, is significantly overexpressed in one-third of OC and fallopian tube cancer (FTC) patients, making it a promising therapeutic target (Scaranti et al., 2020).

Mirvetuximab soravtansine, an ADC comprising a humanized IgG1 mAb targeting FRα, conjugated to the cytotoxin DM4 via a cleavable linker (sulfo-SPDB), with a DAR of approximately 3.5 (Nerone et al., 2022; Heo, 2023). In 2022, the FDA granted approval for mirvetuximab soravtansine to treat FRα-positive PROC in patients who had undergone 1 to 3 prior therapies (Heo, 2023).

In the phase III MIRASOL trial (NCT04209855) involving patients with PROC, mirvetuximab soravtansine demonstrated superior efficacy compared to investigator-selected chemotherapy, reducing the risk of death by 33% and the risk of tumor progression by 35%, along with a lower incidence of Grade≥3 TRAEs and reduced treatment discontinuation rates. Based on these promising results, mirvetuximab soravtansine received approval from the FDA in 2022 for treating FRα-positive PROC, FTC, or primary peritoneal cancer (Heo, 2023; Dilawari et al., 2023; Matulonis et al., 2023). Additionally, the therapeutic potential of mirvetuximab soravtansine has been expanded to other FRα-positive cancers, including endometrial cancer and BC, further enhancing its clinical importance (Kong and Zheng, 2025).

### 5.7 Cetuximab sarotalocan (Akalux®, Rakuten Medical)

**FIGURE 5:**
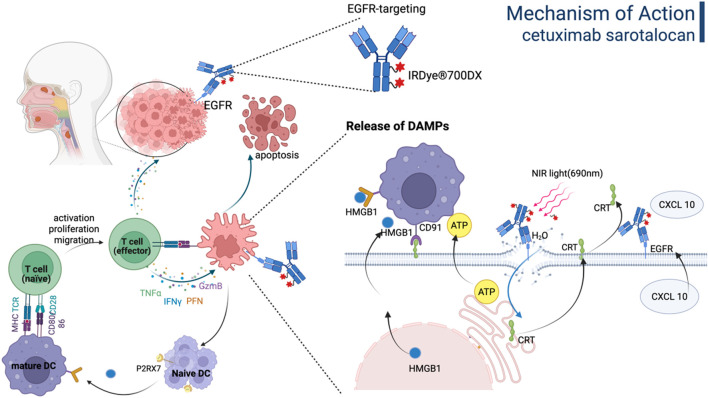
The mechanism of action of Cetuximab sarotalocan involves the direct eradication of tumor cells and immunogenic cell death driven by the release of DAMPs. Figure was created by Biorender.com.

Cetuximab sarotalocan, as the first photoimmunotherapy ADC globally, consists of EGFR-targeting cetuximab conjugated to the photosensitizer IRDye700. Cetuximab is a chimeric IgG1 mAb against EGFR and has been approved for the treatment of colorectal cancer and head and neck cancer. The payload, IRDye700, is a water-soluble silicon phthalocyanine derivative that exhibits sensitivity to red visible light. The average DAR varied between 1.3 and 3.8. Cetuximab sarotalocan selectively binds to EGFR expressed on the tumor cell membrane, followed by irradiation of the tumor site with near-infrared light, which activates the phototoxic effects of IRDye700 to selectively eliminate tumor cells while preserving surrounding normal tissues (Maruoka et al., 2021; Gomes-da-Silva et al., 2020). As illustrated in Figure 5, cetuximab sarotalocan can induce ICD by necrotic disruption of the plasma membrane and the subsequent release of intracellular components, including tumor antigens and DAMPs, thereby activating a robust anti-cancer immune response capable of targeting metastases beyond the irradiated field and eliciting an abscopal effect, which further amplify the anti-tumor effects (Gomes-da-Silva et al., 2020; Gomes-da-Silva et al., 2018; Donohoe et al., 2019; Sato et al., 2018).

In September 2020, the PMDA in Japan approved cetuximab sarotalocan for treating unresectable la/m head and neck squamous cell carcinoma (HNSCC), which was grounded on a phase II clinical trial (Cognetti et al., 2021). Following administration of cetuximab sarotalocan, the tumor area was irradiated with non-thermal red light at 24 h post-administration. Treatment with cetuximab sarotalocan demonstrated an ORR of 28%, including a CR of 14%. The median PFS and median OS were 5.7 months and 9.1 months, respectively. The most frequently observed grade 3 or higher TRAEs included skin reactions (18%), periodontal clefts (12%), and anaphylaxis (3.5%). Cetuximab sarotalocan has not received regulatory approval outside Japan and remains under investigation in global phase III clinical trials (NCT06699212). Other photoimmunotherapy ADCs currently under development globally include TROP-2-IRDye700, antibody C-IRDye700, ramucirumab-IRDye700, and MN-14-700DX, all of which are currently in the preclinical or drug discovery stages.

## 6 Limitations and challenges

As an innovative anti-tumor therapy, ADCs have garnered extensive attention in recent years. Nevertheless, numerous challenges remain in the development and application, which can be categorized into the following aspects.

### 6.1 Adverse effects

To achieve the ideal “intelligent missile” status, ADCs must demonstrate tumor-specific targeting across four critical stages: (1) the antigen target should be exclusively expressed on tumor cells; (2) the ADC should exclusively enter tumor cells through target-independent mechanisms; (3) the linker should be selectively cleaved within the TME or tumor cells; and (4) tumor cells must exhibit significantly higher sensitivity to the cytotoxic payload compared to normal cells. Non-specificity in any of these stages may result in off-target effects, thereby inducing undesirable toxicity (Mahalingaiah et al., 2019). For example, T-DXd, which releases DXd upon cleavage by cathepsin in non-target tissues, leading to an incidence rate of interstitial lung disease ranging from 12% to 19% (Li R. et al., 2024). Depatuxizumab mafodotin (ABT-414) is an ADC directed against EGFR, which utilizes a non-cleavable MMAF payload to reduce bystander effects. Nonetheless, ABT-414 induced significant corneal toxicity due to on-target, off-tumor activity, which ultimately contributed to its termination in phase III trials (Promi et al., 2025).

Additionally, the antibody component of ADCs may induce toxicity through Fc-mediated interactions with normal tissue FcγR or complement activation (Nguyen et al., 2023). For instance, the trastuzumab moiety of T-DM1 mediates platelet phagocytosis via FcγRIIIa binding, thereby causing thrombocytopenia (19%) (Uppal et al., 2015).

### 6.2 Drug resistance

Drug resistance represents one of the most significant challenges in the clinical use of ADC, severely compromising therapeutic efficacy and patient outcomes. The underlying mechanisms of ADC drug resistance are intricate and multifaceted. Tumor cells may reduce the expression of target antigens recognized by ADCs, thereby inhibiting effective binding to tumor cells and escaping drug-induced cytotoxic effects. In HER2-positive BC treated with HER2-targeted ADCs, some patients may experience decreased HER2 expression, leading to impaired drug binding and subsequent resistance (Khoury et al., 2023). Furthermore, ADC resistance can emerge through receptor-mediated endocytosis of target antigens, abnormal lysosomal cleavage processes, and enhanced efflux of the drug-antibody complex. Dysfunctions in the endocytic pathway and lysosomal abnormalities significantly contribute to ADC resistance. For example, caveolin-1-mediated endocytosis could impede ADC entry into lysosomes for degradation (Sung et al., 2018). Loss of the lysosomal transporter SLC46A3 or increased lysosomal pH (which reduces enzyme activity) can further hinder ADC degradation, such as the failure to effectively release cytotoxic payloads following T-DM1 internalization (Ríos-Luci et al., 2017). Abnormal expression of transporters may also enhance ADC efflux, contributing to drug resistance (Takegawa et al., 2017). Additionally, tumor cells may activate alternative signaling pathways to bypass ADC targets, sustaining cell growth and proliferation (Jiang et al., 2024). During HER2-targeted ADC therapy, activation of pathways like PI3K/AKT/mTOR can decrease cellular sensitivity to the drug (Endo et al., 2018).

### 6.3 Pharmacokinetic complexity

Following administration, ADCs circulate systemically primarily in three forms: intact ADCs, naked antibodies, and free payloads. Their relative proportions change dynamically through processes such as target binding, internalization, and dissociation. Due to the typically longer half-lives of intact ADCs and naked antibodies compared with traditional small-molecule drugs—further complicated by interpatient variability—developing pharmacokinetic and pharmacodynamic models that accurately characterize the clinical behavior of ADCs and guide the design of novel agents remains challenging. Owing to their complex pharmacological properties, ADCs exhibit a narrow therapeutic window, necessitating precise dose control. Furthermore, pharmacokinetic variability may lead to considerable differences in efficacy and toxicity among patients. Therefore, developing personalized treatment approaches based on individual patient profiles is essential to enhance therapeutic outcomes and minimize adverse effects (Michael et al., 2024).

## 7 Next-generation ADCs

The next-generation ADCs emphasizes synergistic optimization of four core constituent elements, including antibody engineering, linker chemistry, payload innovation, and conjugation technology. Critical advancement vectors can be conceptualized as:

Future direction focuses on developing tumor specific antigens, such as Claudin 18.2 as well as antigens within the TME or vasculature system (Zhou et al., 2024; Shin et al., 2023; Fu et al., 2019). Moreover, bispecific ADCs represent a strategic evolution in ADC technology by employing dual targeting mechanisms (Gu et al., 2024). BL-B01D1, a first-in-class EGFR/HER3-targeting bispecific ADC, achieved ORR of 39.6% in refractory esophageal squamous cell carcinoma (Liu et al., 2025) and 69.2% in non-classical EGFR-mutant NSCLC with median PFS reaching 10.5 months (Yang et al., 2025), while maintaining a favorable safety profile characterized by manageable TRAEs. Another upgradation is conditionally activated ADCs (probody-drug conjugates) aimed at reducing on-target, off-tumor toxicity (Karen et al., 2019). By incorporating self-masking structures or pH-sensitive binding sites into the antibody, the construct remains unable to bind its target in the neutral pH environment of normal tissues, while becoming activated in the acidic TME (pH 5.3–6.7), enabling specific binding to tumor-associated antigens. In clinical trials, ROR2-targeting conditionally activated ADC BA3021 of BioAtla exhibited promising efficacy and tolerability in patients with HPV-positive HNSCC (Hwai Wen et al., 2025). However, Probody technology of CytomX has encountered setbacks with clinical discontinuations, underscoring the need for further optimization and validation in this area (Karen et al., 2019).

The properties of linkers directly influence the therapeutic index, efficacy, safety, and pharmacokinetics of ADCs. Emerging strategies—such as dual-cleavable linkers, bioorthogonal activation systems, and self-assembling linkers—hold promise for achieving unprecedented tumor specificity, controlled release, and broader therapeutic applications, though their clinical safety and efficacy remain unproven (Bargh et al., 2021; António et al., 2021; Xue et al., 2024; Su et al., 2021; Wei et al., 2018; Tong et al., 2024).

Future advancements in payloads will center on improving efficacy, reducing toxicity, and countering resistance. In addition to novel cytotoxic and immunomodulatory agents, current research emphasizes degrader antibody conjugates (DACs), which employ PROTAC technology to selectively degrade target proteins via the ubiquitin–proteasome system, enabling precise tumor cell elimination (Karina et al., 2023). To date, only Orum Therapeutics’ ORM-6151 has entered Phase I clinical trials. ORM-6151 is a novel DAC comprising a highly potent GSPT1 degrader (SMol006) linked to a CD33-directed antibody (OR000283). Upon internalization, the linker releases SMol006, leading to targeted GSPT1 degradation via the proteasome. In blasts from patients with R/R AML, ORM-6151 exhibited picomolar cytotoxicity and outperformed gemtuzumab ozogamicin. Moreover, a single dose as low as 0.1 mg/kg induced robust and sustained antitumor responses in disseminated xenograft models (Palacino et al., 2023). The co-administration of payloads with dual mechanisms, such as microtubule inhibitors combined with DNA-damaging agents, offers a promising strategy to counter tumor heterogeneity and drug resistance, though further optimization of conjugation methods and toxicity profiles is necessary (Gu et al., 2024). Meanwhile, radionuclide-based payloads leverage localized radiation to enhance antitumor efficacy, but their clinical translation requires careful management of dosimetry and safety (Marco et al., 2025). As the first therapeutic radionuclide-drug conjugate (RDC), Lutathera [(Gesthalter et al., 2022)Lu-Dotatate] targets somatostatin receptors (SSTR) in patients with SSTR-positive gastroenteropancreatic neuroendocrine tumors (GEP-NETs). In the NETTER-1 trial, Lutathera achieved an ORR of 13%, compared to 4% in the control group, reflecting a threefold improvement (Jonathan et al., 2021; Jonathan et al., 2017). Treatment with Lutathera also resulted in a 48% reduction in the risk of death.

The conjugation site significantly influences the stability and pharmacokinetic properties of ADCs: formulations with a higher DAR often exhibit accelerated plasma clearance, while those with lower DAR demonstrate reduced potency. Therefore, developing novel conjugation strategies that enable precise control over the location and number of conjugated small-molecule drugs, while maintaining structural integrity and homogeneity, has become a crucial research direction in the ADC field. New-generation site-specific conjugation strategies comprise enzyme-mediated conjugation (transglutaminase-based), incorporation of non-canonical amino acids, glycoengineering-based conjugation, etc (Tsuchikama and An, 2016). For instance, Strop et al. established a site-specific conjugation approach using a engineered microbial transglutaminase with selective peptide recognition, which targets a genetically incorporated LLQG motif. This strategy allows flexible placement of conjugation sites through intentional insertion of the short peptide into the antibody architecture (Strop et al., 2013). The resulting ADC achieved a uniform DAR of 1.9 while maintaining comparable cytotoxic potency and tolerability. Furthermore, the integration of artificial intelligence (AI) and systems biology establishes a rational drug design pipeline. Machine learning-driven platforms, including AlphaFold-ADC and Schrödinger BioLuminate, enable *de novo* antibody paratope modeling, linker-payload compatibility prediction, and physiologically based pharmacokinetic simulations, reducing preclinical development timelines from 24 to 14 months while increasing clinical trial success rates 3.2-fold (Chen L. et al., 2025; Ma et al., 2025).

In addition, the combination of ADC with other treatment approaches represents a pivotal advancement in cancer therapy. More specifically, when combined with immunotherapy, ADCs can promote the release of tumor antigens and boost T-cell activation through immune checkpoint inhibitors, leading to a synergistic outcome. For example, the combination of T-DXd and pembrolizumab achieved an ORR of 61.5% in HER2-positive gastroesophageal cancer ^264^. Moreover, integrating ADCs with targeted therapies can successfully inhibit drug resistance pathways, as demonstrated using T-DXd alongside neratinib in BC resistant to T-DM1 ^265^. Additionally, when used in combination with chemotherapy, ADCs are more effective due to better penetration and minimized toxicity. Furthermore, current studies are investigating the possibilities of combining ADCs with novel treatments like CAR-T cell therapy, which could amplify anti-tumor responses via multimodal synergy in the future.

## 8 Discussion

As of now, over 400 ADC candidates remain under development. Several ADC candidates are currently in phase III clinical trials (Table 4), undergoing thorough scientific evaluation and extensive clinical testing to confirm the safety and efficacy.

**TABLE 4:** Detailed information regarding selected ADC candidates currently in phase III clinical trials.

Name	Target	Linker	Payload type	DAR	Representative indication	Clinicaltrial NO.
Trastuzumab duocarmazine	HER2	Mc-PEG2-Val-Cit-PABA-Cyc	DNA-damaging agent	2.8	mBC	NCT03262935
TQB-2102	HER2	enzyme-cleavable linker	TOP1 inhibitor	5.8	BC	NCT06561607
DB 1303	HER2	Mc-Gly-Gly-Phe-Gly	TOP1 inhibitor	8	mBC	NCT06018337
Trastuzumab vedotin	HER2	Mc-Val-Cit-PABC	Tubulin binder	3.8	HER2^+^ mBC	NCT04924699
					HER2^+^ mUC	NCT05754853
Trastuzumab envedotin	HER2	PEG2-Val-Cit-PABC	Tubulin binder	2	HER2^+^ BC	NCT06313086 NCT05901935
Anvatabart opadotina	HER2	hydroxylamine-PEG4	Tubulin binder	1.9	HER2^+^ BC	NCT05426486
Caxmotabart entudotin	HER2	geranyl ketone pyrophosphate oxime ligation	Tubulin binder	2	BC	NCT05755048
BL-M07D1	HER2	Mc-Gly-Gly-Phe-Gly	TOP1 inhibitor	8	HER2^-^ low BC	NCT06957886
Trastuzumab botidotin	HER2	Mc-Gly-Gly-Phe-Gly	Tubulin binder	2	HER2^+^ mBC	NCT06968585
IBI-354	HER2	Mc-Gly-Gly-Phe-Gly	TOP1 inhibitor	8	OC/FTC/PPC	NCT06834672
SYS-6010	EGFR	Mc-Gly-Gly-Phe-Gly	TOP1 inhibitor	8	EGFR^+^ mNSCLC	NCT06927986
Becotatug vedotin	EGFR	Mc-Val-Cit-PABC	Tubulin binder	3.8	HNSCC	NCT05751512
Depatuxizumab mafodotin	EGFR	Maleimidocaproyl	Tubulin binder	3.8	GBM	NCT02573324
Luveltamab tazevibulin	FRα	Val-Cit-PABA	Tubulin binder	4	OC/FTC/PPC	NCT05870748
Rinatabart sesutecan	FRα	Cys-11	TOP1 inhibitor	8	PROC	NCT06619236
BAT8006	FRα	Mc-Gly-Gly-Phe-Gly	TOP1 inhibitor	8	FRα^+^ OC/FTC/PPC	CTR20251345
Telisotuzumab adizutecan	MET	Val-Ala	TOP1 inhibitor	—	mCRC	NCT06614192
MHB088C	CD276	Mc-Gly-Gly-Phe-Gly	TOP1 inhibitor	4	SCLC	NCT06954246
Ifinatamab deruxtecan	CD276	Mc-Gly-Gly-Phe-Gly	TOP1 inhibitor	6	ESCC	NCT06644781
					SCLC	NCT06203210
HS-20093	CD276	Mc-Gly-Gly-Phe-Gly	TOP1 inhibitor	4	osteosarcoma	NCT06935409
Patritumab deruxtecan	HER3	mc-Gly-Gly-Phe-Gly	TOP1 inhibitor	8	NSCLC	NCT05338970
Tusamitamab ravtansine	CEACAM5	SPDB	Tubulin binder	3–4	NSCLC	NCT04154956
FDA018	TROP2	PH-sensitive cleavable linkers	TOP1 inhibitor	7.6	TNBC	NCT06519370
HS-20089	B7-H4	Mc-Gly-Gly-Phe-Gly	TOP1 inhibitor	6	OC	NCT06855069
Sonesitatug vedotin	CLDN18.2	Mc-Val-Cit-PABC	Tubulin binder	—	GC/GEJC	NCT06346392
Sigvotatug vedotin	ITGB6	Mc-Val-Cit-PABC	Tubulin binder	4	NSCLC	NCT06758401 NCT06012435
Zilovertamab vedotin	ROR1	Mc-Val-Cit-PABC	Tubulin binder	4	DLBCL	NCT05139017
						NCT06717347
Raludotatug deruxtecan	CDH6	Mc-Gly-Gly-Phe-Gly	TOP1 inhibitor	8	Solid Cancer	NCT06161025

Abbreviation: FTC, fallopian tube cancer; PPC, primary peritoneal carcinoma; PC, pancreatic cancer; HNSCC, head and neck squamous cell carcinoma; GBM, glioblastoma; mCRC, metastatic colorectal cancer; SCLC, small cell lung cancer; ESCC, esophageal squamous cell carcinoma.

ADCs represent a major advance in targeted therapy; however, they still face several clinical challenges. It is essential to recognize that an ADC is an integrated system rather than a mere assembly of modular components. The antibody, target antigen, linker, payload, and DAR are interconnected and constrained. A suboptimal design in any component can be amplified across the entire system. As of now, a total of 128 drug candidates have been discontinued from development. A notable example is REGN5093-M114, a bispecific ADC designed to target two distinct epitopes of the MET receptor and conjugated to the maytansinoid payload M24. However, its phase I/II clinical trial in patients with MET-overexpressing NSCLC was discontinued due to insufficient clinical efficacy (Drilon et al., 2022). SBT6050 is an ISAC that targets HER2 with a linked TLR8 agonist to activate myeloid cells in tumors exhibiting moderate-to-high HER2 expression (Metz et al., 2020). Interim results from its phase I/Ib trial showed that among 14 evaluable patients, there was 1 PR, 3 cases of SD, and 10 cases of PD, yielding an ORR of only 7% (Klempner et al., 2021). These discouraging results underscore the challenges of eliciting antitumor responses through immune activation alone. Agonist ADCs also face inherent obstacles in balancing potency and toxicity, making it difficult to enhance efficacy within a safe therapeutic window. Furthermore, innovation in combination therapies must also consider the balance between additive efficacy and safety. In the phase I trial of rovalpituzumab tesirine (Rova-T), the first DLL3-targeting ADC for small cell lung cancer (SCLC), patients with DLL3-high expression showed an ORR of 31%, a median PFS of 4.6 months, and a median OS of 5.8 months (Christine et al., 2021). However, the combination of Rova-T with nivolumab ± ipilimumab, while achieving an ORR of 30%, was associated with grade 3 or higher adverse events in 92% of patients, leading to early termination of the trial due to poor safety and tolerability (Jyoti et al., 2021).

Nevertheless, these failures are not endpoints but rather starting points for optimizing next-generation ADCs. Continuous improvements in antibody engineering, linker stability, and payload technology have led to significant enhancements in the efficacy and safety profiles of ADCs. With ongoing technological advances and accumulating clinical experience, next-generation ADCs are expected to play an increasingly important role in cancer therapy, offering renewed therapeutic potential for patients.

## References

[B1] Ahn M.-J. Tanaka K. Paz-Ares L. Cornelissen R. Girard N. Pons-Tostivint E. (2025). Datopotamab deruxtecan *versus* docetaxel for previously treated advanced or metastatic non-small cell lung cancer: the randomized, open-label phase III TROPION-Lung01 study. J. Clin. Oncol. 43, 260–272. 10.1200/JCO-24-01544 39250535 PMC11771353

[B2] Akaiwa M. Dugal-Tessier J. Mendelsohn B. A. (2020). Antibody-drug conjugate payloads; study of Auristatin derivatives. Chem. Pharm. Bull. (Tokyo) 68, 201–211. 10.1248/cpb.c19-00853 32115527

[B3] Alderuccio J. P. Alencar A. J. Schatz J. H. Kuker R. A. Pongas G. Reis I. M. (2025). Loncastuximab tesirine with rituximab in patients with relapsed or refractory follicular lymphoma: a single-centre, single-arm, phase 2 trial. Lancet Haematol. 12, e23–e34. 10.1016/S2352-3026(24)00345-4 39662486

[B4] Alibhai S. M. H. Leach M. Minden M. D. Brandwein J. (2009). Outcomes and quality of care in acute myeloid leukemia over 40 years. Cancer 115, 2903–2911. 10.1002/cncr.24373 19452536

[B5] Alvarez Secord A. Lewin S. N. Murphy C. G. Cecere S. C. Barquín A. Gálvez-Montosa F. (2025). The efficacy and safety of mirvetuximab soravtansine in FRα-positive, third-line and later, recurrent platinum-sensitive ovarian cancer: the single-arm phase II PICCOLO trial. Ann. Oncol. 36, 321–330. 10.1016/j.annonc.2024.11.011 39617145

[B6] Amadori S. Suciu S. Selleslag D. Aversa F. Gaidano G. Musso M. (2016). Gemtuzumab ozogamicin *versus* best supportive care in older patients with newly diagnosed acute myeloid leukemia unsuitable for intensive chemotherapy: results of the randomized phase III EORTC-GIMEMA AML-19 trial. J. Clin. Oncol. Official J. Am. Soc. Clin. Oncol. 34, 972–979. 10.1200/jco.2015.64.0060 26811524

[B7] Amiri-Kordestani L. Blumenthal G. M. Xu Q. C. Zhang L. Tang S. W. Ha L. (2014). FDA approval: ado-trastuzumab emtansine for the treatment of patients with HER2-positive metastatic breast cancer. Clin. Cancer Res. 20, 4436–4441. 10.1158/1078-0432.CCR-14-0012 24879797

[B8] Angelastro A. Barkhanskiy A. Mattey A. P. Pallister E. G. Spiess R. Goundry W. (2022). Galactose oxidase enables modular assembly of conjugates from native antibodies with high drug-to-antibody ratios. ChemSusChem 15, e202102592. 10.1002/cssc.202102592 34931761 PMC9303943

[B9] António J. P. M. Carvalho J. I. André A. S. Dias J. N. R. Aguiar S. I. Faustino H. (2021). Diazaborines are a versatile platform to develop ROS-responsive antibody drug conjugates. Angew. Chem. Int. Ed. Engl. 60, 25914–25921. 10.1002/anie.202109835 34741376

[B10] Aoyama M. Tada M. Yokoo H. Ito T. Misawa T. Demizu Y. (2024). Linker and conjugation site synergy in antibody-drug conjugates: impacts on biological activity. Bioconjug Chem. 35, 1568–1576. 10.1021/acs.bioconjchem.4c00348 39363433 PMC11488503

[B11] Appelbaum F. R. Bernstein I. D. (2017). Gemtuzumab ozogamicin for acute myeloid leukemia. Blood 130, 2373–2376. 10.1182/blood-2017-09-797712 29021230

[B12] Bakema J. E. Stigter-van Walsum M. Harris J. R. Ganzevles S. H. Muthuswamy A. Houtkamp M. (2024). An antibody-drug conjugate directed to tissue factor shows preclinical antitumor activity in head and neck cancer as a single agent and in combination with chemoradiotherapy. Mol. Cancer Ther. 23, 187–198. 10.1158/1535-7163.MCT-23-0298 37828725

[B13] Ballantyne A. Dhillon S. (2013). Trastuzumab emtansine: first global approval. Drugs 73, 755–765. 10.1007/s40265-013-0050-2 23620199

[B14] Bardia A. Tolaney S. M. Punie K. Loirat D. Oliveira M. Kalinsky K. (2021a). Biomarker analyses in the phase III ASCENT study of sacituzumab govitecan *versus* chemotherapy in patients with metastatic triple-negative breast cancer. Ann. Oncol. 32, 1148–1156. 10.1016/j.annonc.2021.06.002 34116144

[B15] Bardia A. Messersmith W. A. Kio E. A. Berlin J. D. Vahdat L. Masters G. A. (2021b). Sacituzumab govitecan, a Trop-2-directed antibody-drug conjugate, for patients with epithelial cancer: final safety and efficacy results from the phase I/II IMMU-132-01 basket trial. Ann. Oncol. 32, 746–756. 10.1016/j.annonc.2021.03.005 33741442

[B16] Bardia A. Hurvitz S. A. Tolaney S. M. Loirat D. Punie K. Oliveira M. (2021c). Sacituzumab govitecan in metastatic triple-negative breast cancer. N. Engl. J. Med. 384, 1529–1541. 10.1056/NEJMoa2028485 33882206

[B17] Bardia A. Hu X. Dent R. Yonemori K. Barrios C. H. O’Shaughnessy J. A. (2024a). Trastuzumab deruxtecan after endocrine therapy in metastatic breast cancer. N. Engl. J. Med. 391, 2110–2122. 10.1056/NEJMoa2407086 39282896

[B18] Bardia A. Krop I. E. Kogawa T. Juric D. Tolcher A. W. Hamilton E. P. (2024b). Datopotamab deruxtecan in advanced or metastatic HR+/HER2-and triple-negative breast cancer: results from the phase I TROPION-PanTumor01 study. J. Clin. Oncol. 42, 2281–2294. 10.1200/JCO.23.01909 38652877 PMC11210948

[B19] Bardia A. Jhaveri K. Kalinsky K. Pernas S. Tsurutani J. Xu B. (2024c). TROPION-Breast01: Datopotamab deruxtecan vs chemotherapy in pre-treated inoperable or metastatic HR+/HER2-breast cancer. Future Oncol. 20, 423–436. 10.2217/fon-2023-0188 37387213

[B20] Bardia A. Jhaveri K. Im S.-A. Pernas S. De Laurentiis M. Wang S. (2025a). Datopotamab deruxtecan *versus* chemotherapy in previously treated inoperable/Metastatic hormone receptor-positive human epidermal growth factor receptor 2-Negative breast cancer: primary results from TROPION-Breast01. J. Clin. Oncol. 43, 285–296. 10.1200/JCO.24.00920 39265124 PMC11771365

[B21] Bardia A. Jhaveri K. Im S.-A. Pernas S. Laurentiis M. D. Wang S. (2025b). Datopotamab deruxtecan *versus* chemotherapy in previously treated inoperable/metastatic hormone receptor–positive human epidermal growth factor receptor 2–Negative breast cancer: primary results from TROPION-Breast01. J. Clin. Oncol. 43, 285–296. 10.1200/jco.24.00920 39265124 PMC11771365

[B22] Bargh J. D. Isidro-Llobet A. Parker J. S. Spring D. R. (2019). Cleavable linkers in antibody-drug conjugates. Chem. Soc. Rev. 48, 4361–4374. 10.1039/c8cs00676h 31294429

[B23] Bargh J. D. Walsh S. J. Ashman N. Isidro-Llobet A. Carroll J. S. Spring D. R. (2021). A dual-enzyme cleavable linker for antibody-drug conjugates. Chem. Commun. (Camb) 57, 3457–3460. 10.1039/d1cc00957e 33687404

[B24] Bartlett N. L. Hahn U. Kim W.-S. Fleury I. Laribi K. Bergua J.-M. (2025). Brentuximab vedotin combination for relapsed diffuse large B-Cell lymphoma. J. Clin. Oncol. 43, 1061–1072. 10.1200/jco-24-02242 39772655 PMC11936473

[B25] Ben-Zvi B. Lian C. Brusco M. F. Diao T. (2024). Tunable and photoactivatable mimics of calicheamicin γ1 for DNA cleavage. J. Am. Chem. Soc. 146, 25416–25421. 10.1021/jacs.4c07754 39248674 PMC11421022

[B26] Brave M. H. Maguire W. F. Weinstock C. Zhang H. Gao X. Li F. (2024). FDA approval summary: Enfortumab Vedotin plus pembrolizumab for locally advanced or metastatic urothelial carcinoma. Clin. Cancer Res. 30, 4815–4821. 10.1158/1078-0432.CCR-24-1393 39230571 PMC11530298

[B27] Brinkman J. A. Liu Y. Kron S. J. (2021). Small-molecule drug repurposing to target DNA damage repair and response pathways. Semin. Cancer Biol. 68, 230–241. 10.1016/j.semcancer.2020.02.013 32113999 PMC7483256

[B28] Bross P. F. Beitz J. Chen G. Chen X. H. Duffy E. Kieffer L. (2001). Approval summary: Gemtuzumab ozogamicin in relapsed acute myeloid leukemia. Clin. Cancer Res. 7, 1490–1496. 11410481

[B29] Budde L. E. Olszewski A. J. Assouline S. Lossos I. S. Diefenbach C. Kamdar M. (2024). Mosunetuzumab with polatuzumab vedotin in relapsed or refractory aggressive large B cell lymphoma: a phase 1b/2 trial. Nat. Med. 30, 229–239. 10.1038/s41591-023-02726-5 38072960 PMC10803244

[B30] Burger J. A. Wiestner A. (2018). Targeting B cell receptor signalling in cancer: preclinical and clinical advances. Nat. Rev. Cancer 18, 148–167. 10.1038/nrc.2017.121 29348577

[B31] Caimi P. F. Ai W. Alderuccio J. P. Ardeshna K. M. Hamadani M. Hess B. (2021). Loncastuximab tesirine in relapsed or refractory diffuse large B-cell lymphoma (LOTIS-2): a multicentre, open-label, single-arm, phase 2 trial. Lancet Oncol. 22, 790–800. 10.1016/S1470-2045(21)00139-X 33989558

[B32] Camidge D. R. Bar J. Horinouchi H. Goldman J. Moiseenko F. Filippova E. (2024). Telisotuzumab Vedotin monotherapy in patients with previously treated c-Met protein-overexpressing advanced nonsquamous EGFR-wildtype non-small cell lung cancer in the phase II LUMINOSITY trial. J. Clin. Oncol. 42, 3000–3011. 10.1200/JCO.24.00720 38843488 PMC11361350

[B33] Cao A. T. Law C.-L. Gardai S. J. Heiser R. A. (2017). Abstract 5588: Brentuximab vedotin-driven immunogenic cell death enhances antitumor immune responses, and is potentiated by PD1 inhibition *in vivo* . Cancer Res. 77, 5588. 10.1158/1538-7445.Am2017-5588

[B34] Challita-Eid P. M. Satpayev D. Yang P. An Z. Morrison K. Shostak Y. (2016). Enfortumab vedotin antibody-drug conjugate targeting Nectin-4 is a highly potent therapeutic agent in multiple preclinical cancer models. Cancer Res. 76, 3003–3013. 10.1158/0008-5472.CAN-15-1313 27013195

[B35] Chang E. Weinstock C. Zhang L. Charlab R. Dorff S. E. Gong Y. (2021). FDA approval summary: Enfortumab vedotin for locally advanced or metastatic urothelial carcinoma. Clin. Cancer Res. 27, 922–927. 10.1158/1078-0432.CCR-20-2275 32962979

[B36] Chen Y.-F. Xu Y.-Y. Shao Z.-M. Yu K.-D. (2023). Resistance to antibody-drug conjugates in breast cancer: mechanisms and solutions. Cancer Commun. (Lond) 43, 297–337. 10.1002/cac2.12387 36357174 PMC10009672

[B37] Chen Y. Jia K. Xie Y. Yuan J. Liu D. Jiang L. (2025a). The current landscape of gastric cancer and gastroesophageal junction cancer diagnosis and treatment in China: a comprehensive nationwide cohort analysis. J. Hematol. and Oncol. 18, 42. 10.1186/s13045-025-01698-y 40234884 PMC12001465

[B38] Chen L. Li B. Chen Y. Lin M. Zhang S. Li C. (2025b). ADCNet: a unified framework for predicting the activity of antibody-drug conjugates. Brief. Bioinform 26, bbaf228. 10.1093/bib/bbaf228 40421657 PMC12107246

[B39] Cheng Y. Yuan X. Tian Q. Huang X. Chen Y. Pu Y. (2022). Preclinical profiles of SKB264, a novel anti-TROP2 antibody conjugated to topoisomerase inhibitor, demonstrated promising antitumor efficacy compared to IMMU-132. Front. Oncol. 12, 951589. 10.3389/fonc.2022.951589 36620535 PMC9817100

[B40] Choong G. M. Cullen G. D. O'Sullivan C. C. (2020). Evolving standards of care and new challenges in the management of HER2-positive breast cancer. CA Cancer J. Clin. 70, 355–374. 10.3322/caac.21634 32813307

[B41] Christine L. H. Timothy F. B. Afshin D. Daniel M. Patrick J W. Martina M K. (2021). A phase 1 study evaluating rovalpituzumab tesirine in frontline treatment of patients with extensive-stage SCLC. J. Thorac. Oncol. official Publ. Int. Assoc. Study Lung Cancer 16, 1582–1588. 10.1016/j.jtho.2021.06.022 34242790

[B42] Cognetti D. M. Johnson J. M. Curry J. M. Kochuparambil S. T. McDonald D. Mott F. (2021). Phase 1/2a, open-label, multicenter study of RM-1929 photoimmunotherapy in patients with locoregional, recurrent head and neck squamous cell carcinoma. Head. Neck 43, 3875–3887. 10.1002/hed.26885 34626024 PMC9293150

[B43] Coleman R. L. Lorusso D. Gennigens C. González-Martín A. Randall L. Cibula D. (2021). Efficacy and safety of tisotumab vedotin in previously treated recurrent or metastatic cervical cancer (innovaTV 204/GOG-3023/ENGOT-cx6): a multicentre, open-label, single-arm, phase 2 study. Lancet Oncol. 22, 609–619. 10.1016/S1470-2045(21)00056-5 33845034

[B44] Conilh L. Sadilkova L. Viricel W. Dumontet C. (2023). Payload diversification: a key step in the development of antibody-drug conjugates. J. Hematol. Oncol. 16, 3. 10.1186/s13045-022-01397-y 36650546 PMC9847035

[B45] Cortés J. Kim S.-B. Chung W.-P. Im S.-A. Park Y. H. Hegg R. (2022). Trastuzumab deruxtecan *versus* trastuzumab emtansine for breast cancer. N. Engl. J. Med. 386, 1143–1154. 10.1056/NEJMoa2115022 35320644

[B46] Cowan A. J. Green D. J. Kwok M. Lee S. Coffey D. G. Holmberg L. A. (2022). Diagnosis and management of multiple myeloma: a review. JAMA 327, 464–477. 10.1001/jama.2022.0003 35103762

[B47] De S. K. (2022). Tisotumab vedotin: the first FDA-approved antibody-drug conjugate for cervical cancer. Anticancer Agents Med. Chem. 22, 2808–2810. 10.2174/1871520622666220421095240 35593349

[B48] de Bever L. Popal S. van Schaik J. Rubahamya B. van Delft F. L. Thurber G. M. (2023). Generation of DAR1 antibody-drug conjugates for ultrapotent payloads using tailored GlycoConnect technology. Bioconjug Chem. 34, 538–548. 10.1021/acs.bioconjchem.2c00611 36857521 PMC10020967

[B49] de Bono J. S. Concin N. Hong D. S. Thistlethwaite F. C. Machiels J.-P. Arkenau H.-T. (2019). Tisotumab vedotin in patients with advanced or metastatic solid tumours (InnovaTV 201): a first-in-human, multicentre, phase 1-2 trial. Lancet Oncol. 20, 383–393. 10.1016/S1470-2045(18)30859-3 30745090

[B50] de Goeij B. E. C. G. Satijn D. Freitag C. M. Wubbolts R. Bleeker W. K. Khasanov A. (2015). High turnover of tissue factor enables efficient intracellular delivery of antibody-drug conjugates. Mol. Cancer Ther. 14, 1130–1140. 10.1158/1535-7163.MCT-14-0798 25724665

[B51] Deeks E. D. (2019). Polatuzumab vedotin: first global approval. Drugs 79, 1467–1475. 10.1007/s40265-019-01175-0 31352604 PMC6794237

[B52] DeVita V. T. Rosenberg S. A. (2012). Two hundred years of cancer research. N. Engl. J. Med. 366, 2207–2214. 10.1056/NEJMra1204479 22646510 PMC6293471

[B53] Diéras V. Miles D. Verma S. Pegram M. Welslau M. Baselga J. (2017). Trastuzumab emtansine *versus* capecitabine plus lapatinib in patients with previously treated HER2-positive advanced breast cancer (EMILIA): a descriptive analysis of final overall survival results from a randomised, open-label, phase 3 trial. Lancet Oncol. 18, 732–742. 10.1016/S1470-2045(17)30312-1 28526536 PMC5531181

[B54] Dilawari A. Shah M. Ison G. Gittleman H. Fiero M. H. Shah A. (2023). FDA approval summary: mirvetuximab soravtansine-gynx for FRα-Positive, platinum-resistant ovarian cancer. Clin. Cancer Res. 29, 3835–3840. 10.1158/1078-0432.CCR-23-0991 37212825 PMC10592645

[B55] Dimopoulos M. A. Hungria V. T. M. Radinoff A. Delimpasi S. Mikala G. Masszi T. (2023). Efficacy and safety of single-agent belantamab mafodotin *versus* pomalidomide plus low-dose dexamethasone in patients with relapsed or refractory multiple myeloma (DREAMM-3): a phase 3, open-label, randomised study. Lancet Haematol. 10, e801–e812. 10.1016/S2352-3026(23)00243-0 37793771

[B56] Donohoe C. Senge M. O. Arnaut L. G. Gomes-da-Silva L. C. (2019). Cell death in photodynamic therapy: from oxidative stress to anti-tumor immunity. Biochim. Biophys. Acta Rev. Cancer 1872, 188308. 10.1016/j.bbcan.2019.07.003 31401103

[B57] Drilon A. E. Awad M. M. Gadgeel S. M. Villaruz L. C. Sabari J. K. Perez J. (2022). A phase 1/2 study of REGN5093-M114, a METxMET antibody-drug conjugate, in patients with mesenchymal epithelial transition factor (MET)-Overexpressing NSCLC. J. Clin. Oncol. 40, TPS8593. 10.1200/JCO.2022.40.16_suppl.TPS8593

[B58] Endo Y. Shen Y. Youssef L. A. Mohan N. Wu W. J. (2018). T-DM1-resistant cells gain high invasive activity *via* EGFR and integrin cooperated pathways. MAbs 10, 1003–1017. 10.1080/19420862.2018.1503904 30130447 PMC6260067

[B59] Falini B. Tiacci E. (2024). Hairy-cell leukemia. N. Engl. J. Med. 391, 1328–1341. 10.1056/NEJMra2406376 39383460

[B60] Fang W. Cheng Y. Chen Z. Wang W. Yin Y. Li Y. (2023). SKB264 (TROP2-ADC) for the treatment of patients with advanced NSCLC: efficacy and safety data from a phase 2 study. J. Clin. Oncol. 41, 9114. 10.1200/JCO.2023.41.16_suppl.9114

[B61] Fernandez H. F. Sun Z. Yao X. Litzow M. R. Luger S. M. Paietta E. M. (2009). Anthracycline dose intensification in acute myeloid leukemia. N. Engl. J. Med. 361, 1249–1259. 10.1056/NEJMoa0904544 19776406 PMC4480917

[B62] Francisco J. A. Cerveny C. G. Meyer D. L. Mixan B. J. Klussman K. Chace D. F. (2003). cAC10-vcMMAE, an anti-CD30-monomethyl Auristatin E conjugate with potent and selective antitumor activity. Blood 102, 1458–1465. 10.1182/blood-2003-01-0039 12714494

[B63] Fu Y. Urban D. J. Nani R. R. Zhang Y.-F. Li N. Fu H. (2019). Glypican-3-Specific antibody drug conjugates targeting hepatocellu lar carcinoma. Hepatology 70, 563–576. 10.1002/hep.30326 30353932 PMC6482108

[B64] Fu Z. Li S. Han S. Shi C. Zhang Y. (2022). Antibody drug conjugate: the “biological missile” for targeted cancer therapy. Signal Transduct. Target Ther. 7, 93. 10.1038/s41392-022-00947-7 35318309 PMC8941077

[B65] Fuh F. K. Looney C. Li D. Poon K. A. Dere R. C. Danilenko D. M. (2017). Anti-CD22 and anti-CD79b antibody-drug conjugates preferentially target proliferating B cells. Br. J. Pharmacol. 174, 628–640. 10.1111/bph.13697 28009435 PMC5368047

[B66] Fujiwara Y. Kenmotsu H. Yamamoto N. Shimizu T. Yonemori K. Ocampo C. (2021). Phase 1 study of telisotuzumab vedotin in Japanese patients with advanced solid tumors. Cancer Med. 10, 2350–2358. 10.1002/cam4.3815 33675179 PMC7982615

[B67] Gao H.-F. Li W. Wu Z. Dong J. Cao Y. Zhao Y. (2025). De-escalated neoadjuvant taxane plus trastuzumab and pertuzumab with or without carboplatin in HER2-positive early breast cancer (neoCARHP): a multicentre, open-label, randomised, phase 3 trial. J. Clin. Oncol. 43, LBA500. 10.1200/JCO.2025.43.17_suppl.LBA500

[B68] Gardai S. J. Epp A. Law C.-L. (2015). Abstract 2469: Brentuximab vedotin-mediated immunogenic cell death. Cancer Res. 75, 2469. 10.1158/1538-7445.Am2015-2469

[B69] Gesthalter Y. Smyth R. Sullivan D. (2022). Treatment of advanced-stage non-small cell lung cancer. Am. J. Respir. Crit. Care Med. 205, P9–P10. 10.1164/rccm.2055P9 35230219

[B70] Gianni L. Pienkowski T. Im Y.-H. Roman L. Tseng L.-M. Liu M.-C. (2012). Efficacy and safety of neoadjuvant pertuzumab and trastuzumab in women with locally advanced, inflammatory, or early HER2-positive breast cancer (NeoSphere): a randomised multicentre, open-label, phase 2 trial. Lancet Oncol. 13, 25–32. 10.1016/S1470-2045(11)70336-9 22153890

[B71] Goffart S. Hangas A. Pohjoismäki J. L. O. (2019). Twist and turn-topoisomerase functions in mitochondrial DNA maintenance. Int. J. Mol. Sci. 20, 2041. 10.3390/ijms20082041 31027213 PMC6514783

[B72] Goldmacher V. S. Kovtun Y. V. (2011). Antibody-drug conjugates: using monoclonal antibodies for delivery of cytotoxic payloads to cancer cells. Ther. Deliv. 2, 397–416. 10.4155/tde.10.98 22834009

[B73] Gomes-da-Silva L. C. Zhao L. Bezu L. Zhou H. Sauvat A. Liu P. (2018). Photodynamic therapy with redaporfin targets the endoplasmic reticulum and golgi apparatus. EMBO J. 37, e98354. 10.15252/embj.201798354 29807932 PMC6028029

[B74] Gomes-da-Silva L. C. Kepp O. Kroemer G. (2020). Regulatory approval of photoimmunotherapy: photodynamic therapy that induces immunogenic cell death. Oncoimmunology 9, 1841393. 10.1080/2162402X.2020.1841393 33178498 PMC7595598

[B75] Grever M. R. Blachly J. S. Andritsos L. A. (2014). Hairy cell leukemia: update on molecular profiling and therapeutic advances. Blood Rev. 28, 197–203. 10.1016/j.blre.2014.06.003 25110197

[B76] Gu Y. Wang Z. Wang Y. (2024). Bispecific antibody drug conjugates: making 1+1>2. Acta Pharm. Sin. B 14, 1965–1986. 10.1016/j.apsb.2024.01.009 38799638 PMC11119582

[B77] Gupta S. Loriot Y. Van der Heijden M. S. Bedke J. Valderrama B. P. Kikuchi E. (2025). Enfortumab vedotin plus pembrolizumab *versus* chemotherapy in patients with previously untreated locally advanced or metastatic urothelial cancer (EV-302): patient-reported outcomes from an open-label, randomised, controlled, phase 3 study. Lancet Oncol. 26, 795–805. 10.1016/S1470-2045(25)00158-5 40449498

[B78] Hamilton E. Shastry M. Shiller S. M. Ren R. (2021). Targeting HER2 heterogeneity in breast cancer. Cancer Treat. Rev. 100, 102286. 10.1016/j.ctrv.2021.102286 34534820

[B79] Hartmann R. W. Fahrner R. Shevshenko D. Fyrknäs M. Larsson R. Lehmann F. (2020). Rational design of azastatin as a potential ADC payload with reduced bystander killing. ChemMedChem 15, 2500–2512. 10.1002/cmdc.202000497 33063934 PMC7756782

[B80] Hatschek T. Foukakis T. Bjöhle J. Lekberg T. Fredholm H. Elinder E. (2021). Neoadjuvant trastuzumab, pertuzumab, and docetaxel vs trastuzumab emtansine in patients with ERBB2-Positive breast cancer: a phase 2 randomized clinical trial. JAMA Oncol. 7, 1360–1367. 10.1001/jamaoncol.2021.1932 34165503 PMC8227457

[B81] Heiser R. A. Cao A. T. Zeng W. Ulrich M. Younan P. Anderson M. E. (2024). Brentuximab vedotin-driven microtubule disruption results in endoplasmic reticulum stress leading to immunogenic cell death and antitumor immunity. Mol. Cancer Ther. 23, 68–83. 10.1158/1535-7163.MCT-23-0118 37775098 PMC10762337

[B82] Heo Y.-A. (2023). Mirvetuximab soravtansine: first approval. Drugs 83, 265–273. 10.1007/s40265-023-01834-3 36656533

[B83] Hoffmann R. M. Silvia M. M I. K. Anthony C. Silvia C. Karagiannis S. N. (2018). Antibody structure and engineering considerations for the design and function of antibody drug conjugates (ADCs). OncoImmunology 7. 10.1080/2162402X.2017.1395127 PMC5769674 29375935

[B84] Hohmann T. Dehghani F. (2019). The Cytoskeleton-A complex interacting meshwork. Cells 8, 362. 10.3390/cells8040362 31003495 PMC6523135

[B85] Hwai Wen C. Gerhard F. Jing W. Haizhen L. Charles X. Jian C. (2025). Preclinical development of ozuriftamab vedotin (BA3021), a novel ROR2-specific conditionally active biologic antibody-drug conjugate. mAbs 17, 2490078. 10.1080/19420862.2025.2490078 40202784 PMC11988251

[B86] Indini A. Nigro O. Lengyel C. G. Ghidini M. Petrillo A. Lopez S. (2021). Immune-checkpoint inhibitors in platinum-resistant ovarian cancer. Cancers (Basel) 13, 1663. 10.3390/cancers13071663 33916221 PMC8037571

[B87] Janke C. Magiera M. M. (2020). The tubulin code and its role in controlling microtubule properties and functions. Nat. Rev. Mol. Cell Biol. 21, 307–326. 10.1038/s41580-020-0214-3 32107477

[B88] Jiang M. Li Q. Xu B. (2024). Spotlight on ideal target antigens and resistance in antibody-drug conjugates: strategies for competitive advancement. Drug Resist Updat 75, 101086. 10.1016/j.drup.2024.101086 38677200

[B89] Jin S. Sun Y. Liang X. Gu X. Ning J. Xu Y. (2022). Emerging new therapeutic antibody derivatives for cancer treatment. Signal Transduct. Target Ther. 7, 39. 10.1038/s41392-021-00868-x 35132063 PMC8821599

[B90] Jonathan S. Ghassan E.-H. Edward W. Andrew H. James Y. Beth C. (2017). Phase 3 trial of (177)Lu-Dotatate for midgut neuroendocrine tumors. N. Engl. J. Med. 376, 125–135. 10.1056/NEJMoa1607427 28076709 PMC5895095

[B91] Jonathan R. S. Martyn E C. Pamela L. K. Philippe B. R. Lisa B. Andrew H. (2021). (177)Lu-Dotatate plus long-acting octreotide *versus* high-dose long-acting octreotide in patients with midgut neuroendocrine tumours (NETTER-1): final overall survival and long-term safety results from an open-label, randomised, controlled, phase 3 trial. Lancet. Oncol. 22, 1752–1763. 10.1016/s1470-2045(21)00572-6 34793718

[B92] Junttila T. T. Li G. Parsons K. Phillips G. L. Sliwkowski M. X. (2011). Trastuzumab-DM1 (T-DM1) retains all the mechanisms of action of trastuzumab and efficiently inhibits growth of lapatinib insensitive breast cancer. Breast Cancer Res. Treat. 128, 347–356. 10.1007/s10549-010-1090-x 20730488

[B93] Jyoti M. Petros N. Ticiana L. Jonathan L. Daniel M. Jyoti D P. (2021). A phase 1-2 study of rovalpituzumab tesirine in combination with nivolumab plus or minus ipilimumab in patients with previously treated extensive-stage SCLC. J. Thorac. Oncol. official Publ. Int. Assoc. Study Lung Cancer 16, 1559–1569. 10.1016/j.jtho.2021.02.022 33652156

[B94] Kantarjian H. M. DeAngelo D. J. Stelljes M. Liedtke M. Stock W. Gökbuget N. (2019). Inotuzumab ozogamicin *versus* standard of care in relapsed or refractory acute lymphoblastic leukemia: final report and long-term survival follow-up from the randomized, phase 3 INO-VATE study. Cancer 125, 2474–2487. 10.1002/cncr.32116 30920645 PMC6618133

[B95] Karen A. A. Valentina B. Rachel W H. Aung N. (2019). Probody therapeutics: an emerging class of therapies designed to enhance On-Target effects with reduced off-tumor toxicity for use in immuno-oncology. Clin. cancer Res. official J. Am. Assoc. Cancer Res. 26. 10.1158/1078-0432.Ccr-19-1457 PMC8436251 31601568

[B96] Karina C. Preethi Soundarya S. Markus A Q. Michael M. Harry S. Diane M C. (2023). Antibody-proteolysis targeting chimera conjugate enables selective degradation of receptor-interacting Serine/threonine-protein kinase 2 in HER2+ cell lines. Bioconjug Chem. 34, 2049–2054. 10.1021/acs.bioconjchem.3c00366 37917829 PMC10655034

[B97] Katz J. Janik J. E. Younes A. (2011). Brentuximab vedotin (SGN-35). Clin. Cancer Res. 17, 6428–6436. 10.1158/1078-0432.CCR-11-0488 22003070

[B98] Keam S. J. (2020). Trastuzumab deruxtecan: first approval. Drugs 80, 501–508. 10.1007/s40265-020-01281-4 32144719

[B99] Khera E. Thurber G. M. (2018). Pharmacokinetic and immunological considerations for expanding the therapeutic window of next-generation antibody-drug conjugates. BioDrugs 32, 465–480. 10.1007/s40259-018-0302-5 30132210

[B100] Khoury R. Saleh K. Khalife N. Saleh M. Chahine C. Ibrahim R. (2023). Mechanisms of resistance to antibody-drug conjugates. Int. J. Mol. Sci. 24, 9674. 10.3390/ijms24119674 37298631 PMC10253543

[B101] Klempner S. J. Beeram M. Sabanathan D. Chan A. Hamilton E. Loi S. (2021). 209P interim results of a phase I/Ib study of SBT6050 monotherapy and pembrolizumab combination in patients with advanced HER2-expressing or amplified solid tumors. Ann. Oncol. 32, S450. 10.1016/j.annonc.2021.08.491

[B102] Köhler G. Milstein C. (1975). Continuous cultures of fused cells secreting antibody of predefined specificity. Nature 256, 495–497. 10.1038/256495a0 1172191

[B103] Kong B. Zheng W. (2025). Mirvetuximab soravtansine: current and future applications. J. Hematol. Oncol. 18, 33. 10.1186/s13045-025-01686-2 40102896 PMC11921575

[B104] Kreitman R. J. Pastan I. (2011). Antibody fusion proteins: Anti-CD22 recombinant immunotoxin moxetumomab pasudotox. Clin. Cancer Res. 17, 6398–6405. 10.1158/1078-0432.CCR-11-0487 22003067 PMC3201735

[B105] Kreitman R. J. Dearden C. Zinzani P. L. Delgado J. Karlin L. Robak T. (2018). Moxetumomab pasudotox in relapsed/refractory hairy cell leukemia. Leukemia 32, 1768–1777. 10.1038/s41375-018-0210-1 30030507 PMC6087717

[B106] Lambert J. M. Chari R. V. J. (2014). Ado-trastuzumab emtansine (T-DM1): an antibody-drug conjugate (ADC) for HER2-positive breast cancer. J. Med. Chem. 57, 6949–6964. 10.1021/jm500766w 24967516

[B107] Lambert J. Pautas C. Terré C. Raffoux E. Turlure P. Caillot D. (2019). Gemtuzumab ozogamicin for *de novo* acute myeloid leukemia: final efficacy and safety updates from the open-label, phase III ALFA-0701 trial. Haematologica 104, 113–119. 10.3324/haematol.2018.188888 30076173 PMC6312010

[B108] Lanza F. Maffini E. Rondoni M. Massari E. Faini A. C. Malavasi F. (2020). CD22 expression in B-Cell acute lymphoblastic leukemia: biological significance and implications for inotuzumab therapy in adults. Cancers (Basel) 12, 303. 10.3390/cancers12020303 32012891 PMC7072635

[B109] Lee M. D. Ellestad G. A. Borders D. B. (1991). Calicheamicins: discovery, structure, chemistry, and interaction with DNA. Accounts Chem. Res. 24, 235–243. 10.1021/ar00008a003

[B110] Lenk L. Carlet M. Vogiatzi F. Spory L. Winterberg D. Cousins A. (2021). CD79a promotes CNS-Infiltration and leukemia engraftment in pediatric B-cell precursor acute lymphoblastic leukemia. Commun. Biol. 4, 73. 10.1038/s42003-020-01591-z 33452446 PMC7810877

[B111] Lennerz J. K. Kwak E. L. Ackerman A. Michael M. Fox S. B. Bergethon K. (2011). MET amplification identifies a small and aggressive subgroup of esophagogastric adenocarcinoma with evidence of responsiveness to crizotinib. J. Clin. Oncol. 29, 4803–4810. 10.1200/JCO.2011.35.4928 22042947 PMC3255989

[B112] Li Y. Abudureheiyimu N. Mo H. Guan X. Lin S. Wang Z. (2021). In real life, low-level HER2 expression may be associated with better outcome in HER2-Negative breast cancer: a study of the national cancer center, China. Front. Oncol. 11, 774577. 10.3389/fonc.2021.774577 35111669 PMC8801428

[B113] Li W. Zhang X. Du Y. Zhang Y. Lu J. Hu W. (2022). HER2-targeted advanced metastatic gastric/gastroesophageal junction adenocarcinoma: treatment landscape and future perspectives. Biomark. Res. 10, 71. 10.1186/s40364-022-00416-x 36175985 PMC9524015

[B114] Li Z. Song Z. Hong W. Yang N. Wang Y. Jian H. (2024a). SHR-A1811 (antibody-drug conjugate) in advanced HER2-mutant non-small cell lung cancer: a multicenter, open-label, phase 1/2 study. Signal Transduct. Target Ther. 9, 182. 10.1038/s41392-024-01897-y 39004647 PMC11247081

[B115] Li R. Hua M. Li J. Chen W. Xu L. Meng H. (2024b). The safety of trastuzumab deruxtecan (DS-8201) with a focus on interstitial lung disease And/Or pneumonitis: a systematic review and single-arm meta-analysis. Cancer 130, 2968–2977. 10.1002/cncr.35349 38703012

[B116] Li Z. Wang Y. Sun Y. Wang L. Li X. Sun L. (2025a). Trastuzumab rezetecan, a HER2-directed antibody-drug conjugate, in patients with advanced HER2-mutant non-small-cell lung cancer (HORIZON-Lung): phase 2 results from a multicentre, single-arm study. Lancet Oncol. 26, 437–446. 10.1016/S1470-2045(25)00012-9 40020696

[B117] Li N. Yang L. Zhao Z. Du T. Liang G. Li N. (2025b). Antibody-drug conjugates in breast cancer: current evidence and future directions. Exp. Hematol. Oncol. 14, 41. 10.1186/s40164-025-00632-9 40114224 PMC11924693

[B118] Li J. J. Wang Z. H. Chen L. Zhang W. J. Ma L. X. X. Wu J. (2025c). Efficacy and safety of neoadjuvant SHR-A1811 with or without pyrotinib in women with locally advanced or early HER2-positive breast cancer: a randomized, open-label, phase II trial. Ann. Oncol. 36, 651–659. 10.1016/j.annonc.2025.02.011 40049447

[B119] Linenberger M. L. Hong T. Flowers D. Sievers E. L. Gooley T. A. Bennett J. M. (2001). Multidrug-resistance phenotype and clinical responses to gemtuzumab ozogamicin. Blood 98, 988–994. 10.1182/blood.v98.4.988 11493443

[B120] Liu R. Oldham R. J. Teal E. Beers S. A. Cragg M. S. (2020). Fc-Engineering for modulated effector functions-improving antibodies for cancer treatment, Fc-Engineering Modul. Eff. Functions-Improving Antibodies Cancer Treat. Antibodies (Basel) 9. 64, 10.3390/antib9040064 33212886 PMC7709126

[B121] Liu Y. Han X. Li L. Zhang Y. Huang X. Li G. (2021). Role of Nectin‑4 protein in cancer (review). Int. J. Oncol. 59, 93. 10.3892/ijo.2021.5273 34664682

[B122] Liu C. Liu D. Ji Y. Sun M. Gao S. Ma X. (2025). A bispecific antibody-drug conjugate targeting EGFR and HER3 in metastatic esophageal squamous cell carcinoma: a phase 1b trial. Nat. Med. 10.1038/s41591-025-03792-7 PMC12532689 40640393

[B123] Lonial S. Lee H. C. Badros A. Trudel S. Nooka A. K. Chari A. (2020). Belantamab mafodotin for relapsed or refractory multiple myeloma (DREAMM-2): a two-arm, randomised, open-label, phase 2 study. Lancet Oncol. 21, 207–221. 10.1016/S1470-2045(19)30788-0 31859245

[B124] Lucas A. T. Price L. S. L. Schorzman A. N. Storrie M. Piscitelli J. A. Razo J. (2018). Factors affecting the pharmacology of antibody-drug conjugates. Antibodies (Basel) 7 (1). 10.3390/antib7010010 PMC6698819 31544862

[B125] Lux M. P. Nabieva N. Hartkopf A. D. Huober J. Volz B. Taran F.-A. (2018). Therapy landscape in patients with metastatic HER2-Positive breast cancer: data from the PRAEGNANT real-world breast cancer registry. Cancers (Basel) 11, 10. 10.3390/cancers11010010 30577662 PMC6357172

[B126] Ma P. C. Maulik G. Christensen J. Salgia R. (2003). c-Met: structure, functions and potential for therapeutic inhibition. Cancer Metastasis Rev. 22, 309–325. 10.1023/a:1023768811842 12884908

[B127] Ma D. Dai L.-J. Wu X.-R. Liu C.-L. Zhao S. Zhang H. (2025). Spatial determinants of antibody-drug conjugate SHR-A1811 efficacy in neoadjuvant treatment for HER2-positive breast cancer. Cancer Cell 43, 1061–1075.e7. 10.1016/j.ccell.2025.03.017 40215979

[B128] Maddocks K. (2021). The era of CD19-directed therapy in diffuse large B-cell lymphoma. Lancet Oncol. 22, 741–742. 10.1016/S1470-2045(21)00191-1 33989556

[B129] Mahalingaiah P. K. Ciurlionis R. Durbin K. R. Yeager R. L. Philip B. K. Bawa B. (2019). Potential mechanisms of target-independent uptake and toxicity of antibody-drug conjugates. Pharmacol. Ther. 200, 110–125. 10.1016/j.pharmthera.2019.04.008 31028836

[B130] Mantaj J. Jackson P. J. Rahman K. M. Thurston D. E. (2017). From anthramycin to pyrrolobenzodiazepine (PBD)-Containing antibody-drug conjugates (ADCs). Angew. Chem. Int. Ed. Engl. 56, 462–488. 10.1002/anie.201510610 27862776 PMC5215561

[B131] Marco M. Tony G. Jacqueline M. Dario N. Samuele C. Sebastian O. (2025). Small organic carbonic anhydrase IX ligands from DNA-Encoded chemical libraries for tumor-targeted delivery of radionuclides. J. Am. Chem. Soc. 147, 18230–18239. 10.1021/jacs.5c05198 40378292

[B132] Markham A. (2020). Belantamab Mafodotin: first approval. Drugs 80, 1607–1613. 10.1007/s40265-020-01404-x 32936437

[B133] Markham A. (2021). Tisotumab vedotin: first approval. Drugs 81, 2141–2147. 10.1007/s40265-021-01633-8 34748188

[B134] Maruoka Y. Wakiyama H. Choyke P. L. Kobayashi H. (2021). Near infrared photoimmunotherapy for cancers: a translational perspective. EBioMedicine 70, 103501. 10.1016/j.ebiom.2021.103501 34332294 PMC8340111

[B135] Matulonis U. A. Lorusso D. Oaknin A. Pignata S. Dean A. Denys H. (2023). Efficacy and safety of mirvetuximab soravtansine in patients with platinum-resistant ovarian cancer with high folate receptor alpha expression: results from the SORAYA study. J. Clin. Oncol. 41, 2436–2445. 10.1200/JCO.22.01900 36716407 PMC10150846

[B136] Mayer R. J. Davis R. B. Schiffer C. A. Berg D. T. Powell B. L. Schulman P. (1994). Intensive postremission chemotherapy in adults with acute myeloid leukemia. Cancer and leukemia group B. N. Engl. J. Med. 331, 896–903. 10.1056/NEJM199410063311402 8078551

[B137] McCurdy A. Reece D. Louzada M. L. White D. Parkin S. Chu M. P. (2024). Belantamab mafodotin, pomalidomide, and dexamethasone for triple class exposed/refractory relapsed multiple myeloma: a subgroup analysis of the ALGONQUIN trial. Blood Cancer J. 14, 155. 10.1038/s41408-024-01135-2 39261451 PMC11391083

[B138] Meric-Bernstam F. Makker V. Oaknin A. Oh D.-Y. Banerjee S. González-Martín A. (2024). Efficacy and safety of trastuzumab deruxtecan in patients with HER2-Expressing solid tumors: primary results from the DESTINY-PanTumor02 phase II trial. J. Clin. Oncol. 42, 47–58. 10.1200/jco.23.02005 37870536 PMC10730032

[B139] Meric-Bernstam F. Alhalabi O. Lisberg A. Drakaki A. Garmezy B. Kogawa T. (2025). Datopotamab deruxtecan (Dato-DXd) in locally advanced/metastatic urothelial cancer: updated results from the phase 1 TROPIONPanTumor01 study. J. Clin. Oncol. 43, 663. 10.1200/JCO.2025.43.5_suppl.663

[B140] Metz H. Childs M. Brevik J. Winship D. Brender T. Comeau M. (2020). SBT6050, a HER2-directed TLR8 therapeutic, as a systemically administered, tumor-targeted human myeloid cell agonist. J. Clin. Oncol. 38, 3110. 10.1200/JCO.2020.38.15_suppl.3110

[B141] Michael Z. L. Douglas D L. Shang-Chiung C. Zao L. Amrita V K. Chunze L. (2024). Translational PK/PD framework for antibody-drug conjugates to inform drug discovery and development. Xenobiotica 54, 543–551. 10.1080/00498254.2024.2351044 38738473

[B142] Mittendorf E. A. Zhang H. Barrios C. H. Saji S. Jung K. H. Hegg R. (2020). Neoadjuvant atezolizumab in combination with sequential nab-paclitaxel and anthracycline-based chemotherapy *versus* placebo and chemotherapy in patients with early-stage triple-negative breast cancer (IMpassion031): a randomised, double-blind, phase 3 trial. Lancet 396, 1090–1100. 10.1016/S0140-6736(20)31953-X 32966830

[B143] Modi S. Saura C. Yamashita T. Park Y. H. Kim S.-B. Tamura K. (2020). Trastuzumab deruxtecan in previously treated HER2-Positive breast cancer. N. Engl. J. Med. 382, 610–621. 10.1056/NEJMoa1914510 31825192 PMC7458671

[B144] Moskowitz C. H. Nademanee A. Masszi T. Agura E. Holowiecki J. Abidi M. H. (2015). Brentuximab vedotin as consolidation therapy after autologous stem-cell transplantation in patients with hodgkin's lymphoma at risk of relapse or progression (AETHERA): a randomised, double-blind, placebo-controlled, phase 3 trial. Lancet 385, 1853–1862. 10.1016/S0140-6736(15)60165-9 25796459

[B145] Motwani M. Panchabhai S. Bar J. Girard N. Bradbury P. Lu S. (2021). P60.12 prevalence of c-Met overexpression (c-Met+) and impact of prior lines of treatment on c-Met protein expression in NSCLC. J. Thorac. Oncol. 16, S1169–S1170. 10.1016/j.jtho.2021.08.633

[B146] Mullard A. (2021). FDA approves ADC therapeutics' loncastuximab tesirine, ushering in a new cytotoxic payload. Nat. Rev. Drug Discov. 20, 414. 10.1038/d41573-021-00082-y 33981088

[B147] Müller P. Martin K. Theurich S. Schreiner J. Savic S. Terszowski G. (2014). Microtubule-depolymerizing agents used in antibody-drug conjugates induce antitumor immunity by stimulation of dendritic cells. Cancer Immunol. Res. 2, 741–755. 10.1158/2326-6066.CIR-13-0198 24916470

[B148] Nadal R. Valderrama B. P. Bellmunt J. (2024). Progress in systemic therapy for advanced-stage urothelial carcinoma. Nat. Rev. Clin. Oncol. 21, 8–27. 10.1038/s41571-023-00826-2 37945764

[B149] Nelson B. E. Hong A. Jana B. (2021). Elucidation of novel molecular targets for therapeutic strategies in urothelial carcinoma: a literature review. Front. Oncol. 11, 705294. 10.3389/fonc.2021.705294 34422659 PMC8374860

[B150] Neri P. Leblay N. Lee H. Gulla A. Bahlis N. J. Anderson K. C. (2024). Just scratching the surface: novel treatment approaches for multiple myeloma targeting cell membrane proteins. Nat. Rev. Clin. Oncol. 21, 590–609. 10.1038/s41571-024-00913-y 38961233

[B151] Nerone M. Grande M. D. Sessa C. Colombo I. (2022). Advancing antibody-drug conjugates in gynecological malignancies: myth or reality? Explor Target Antitumor Ther. 3, 149–171. 10.37349/etat.2022.00077 36046840 PMC9400759

[B152] Nguyen T. D. Bordeau B. M. Balthasar J. P. (2023). Mechanisms of ADC toxicity and strategies to increase ADC tolerability. Cancers (Basel) 15, 713. 10.3390/cancers15030713 36765668 PMC9913659

[B153] O'Reilly M. K. Paulson J. C. (2009). Siglecs as targets for therapy in immune-cell-mediated disease. Trends Pharmacol. Sci. 30, 240–248. 10.1016/j.tips.2009.02.005 19359050 PMC2830709

[B154] Ogitani Y. Hagihara K. Oitate M. Naito H. Agatsuma T. (2016). Bystander killing effect of DS-8201a, a novel anti-human epidermal growth factor receptor 2 antibody-drug conjugate, in tumors with human epidermal growth factor receptor 2 heterogeneity. Cancer Sci. 107, 1039–1046. 10.1111/cas.12966 27166974 PMC4946713

[B155] Okajima D. Yasuda S. Maejima T. Karibe T. Sakurai K. Aida T. (2021). Datopotamab deruxtecan, a novel TROP2-directed antibody-drug conjugate, demonstrates potent antitumor activity by efficient drug delivery to tumor cells. Mol. Cancer Ther. 20, 2329–2340. 10.1158/1535-7163.MCT-21-0206 34413126 PMC9398094

[B156] Okeley N. M. Miyamoto J. B. Zhang X. Sanderson R. J. Benjamin D. R. Sievers E. L. (2010). Intracellular activation of SGN-35, a potent anti-CD30 antibody-drug conjugate. Clin. Cancer Res. 16, 888–897. 10.1158/1078-0432.CCR-09-2069 20086002

[B157] Organ S. L. Tsao M.-S. (2011). An overview of the c-MET signaling pathway. Ther. Adv. Med. Oncol. 3, S7-S19. 10.1177/1758834011422556 22128289 PMC3225017

[B158] Palacino J. Lee P. Jeong H. Kim Y. Song Y. Permpoon U. (2023). Abstract 2700: ORM-6151: a first-in-class CD33-antibody enabled GSPT1 degrader for AML. Cancer Res. 83, 2700. 10.1158/1538-7445.Am2023-2700

[B159] Paz-Ares L. G. Juan-Vidal O. Mountzios G. S. Felip E. Reinmuth N. de Marinis F. (2024). Sacituzumab govitecan *versus* docetaxel for previously treated advanced or metastatic non-small cell lung cancer: the randomized, open-label phase III EVOKE-01 study. J. Clin. Oncol. 42, 2860–2872. 10.1200/JCO.24.00733 38843511 PMC11328920

[B160] Peng Z. Liu T. Wei J. Wang A. He Y. Yang L. (2021). Efficacy and safety of a novel anti-HER2 therapeutic antibody RC48 in patients with HER2-overexpressing, locally advanced or metastatic gastric or gastroesophageal junction cancer: a single-arm phase II study. Cancer Commun. (Lond) 41, 1173–1182. 10.1002/cac2.12214 34665942 PMC8626607

[B161] Petersdorf S. H. Kopecky K. J. Slovak M. Willman C. Nevill T. Brandwein J. (2013). A phase 3 study of gemtuzumab ozogamicin during induction and postconsolidation therapy in younger patients with acute myeloid leukemia. Blood 121, 4854–4860. 10.1182/blood-2013-01-466706 23591789 PMC3682338

[B162] Pfeifer M. Zheng B. Erdmann T. Koeppen H. McCord R. Grau M. (2015). Anti-CD22 and anti-CD79B antibody drug conjugates are active in different molecular diffuse large B-cell lymphoma subtypes. Leukemia 29, 1578–1586. 10.1038/leu.2015.48 25708834

[B163] Polson A. G. Yu S.-F. Elkins K. Zheng B. Clark S. Ingle G. S. (2007). Antibody-drug conjugates targeted to CD79 for the treatment of Non-Hodgkin lymphoma. Blood 110, 616–623. 10.1182/blood-2007-01-066704 17374736

[B164] Powles T. Rosenberg J. E. Sonpavde G. P. Loriot Y. Durán I. Lee J.-L. (2021). Enfortumab vedotin in previously treated advanced urothelial carcinoma. N. Engl. J. Med. 384, 1125–1135. 10.1056/NEJMoa2035807 33577729 PMC8450892

[B165] Powles T. Tagawa S. Vulsteke C. Gross-Goupil M. Park S. H. Necchi A. (2025). Sacituzumab govitecan in advanced urothelial carcinoma: TROPiCS-04, a phase III randomized trial. Ann. Oncol. 36, 561–571. 10.1016/j.annonc.2025.01.011 39934055

[B166] Prince H. M. Kim Y. H. Horwitz S. M. Dummer R. Scarisbrick J. Quaglino P. (2017). Brentuximab vedotin or physician's choice in CD30-positive cutaneous T-cell lymphoma (ALCANZA): an international, open-label, randomised, phase 3, multicentre trial. Lancet 390, 555–566. 10.1016/S0140-6736(17)31266-7 28600132

[B167] Pro B. Advani R. Brice P. Bartlett N. L. Rosenblatt J. D. Illidge T. (2012). Brentuximab vedotin (SGN-35) in patients with relapsed or refractory systemic anaplastic large-cell lymphoma: results of a phase II study. J. Clin. Oncol. 30, 2190–2196. 10.1200/JCO.2011.38.0402 22614995

[B168] Promi S. A. Khalil I. Kulsum U. Islam M. R. Eva I. I. Sayed M. A. (2025). Efficacy and safety of depatuxizumab mafodotin (ABT-414) in EGFR-Amplified glioblastoma: a systematic review and Bayesian network meta-analysis. J. Clin. Oncol. 43, 2060. 10.1200/JCO.2025.43.16_suppl.2060

[B169] Pulte D. Gondos A. Brenner H. (2009). Improvement in survival in younger patients with acute lymphoblastic leukemia from the 1980s to the early 21st century. Blood 113, 1408–1411. 10.1182/blood-2008-06-164863 18974371

[B170] Qu F. Li W. Yin Y. Liu Q. (2024). Abstract PO3-04-05: efficacy and safety of the recombinant humanized anti-HER2 monoclonal antibody-MMAE conjugate RC48-ADC in patients with HER2-positive or HER2-low expressing, metastatic breast cancer: a single-arm phase II study. Cancer Res. 84, PO3–05. 10.1158/1538-7445.Sabcs23-po3-04-05

[B171] Raghav K. Siena S. Takashima A. Kato T. Van den Eynde M. Pietrantonio F. (2024). Trastuzumab deruxtecan in patients with HER2-positive advanced colorectal cancer (DESTINY-CRC02): primary results from a multicentre, randomised, phase 2 trial. Lancet Oncol. 25, 1147–1162. 10.1016/S1470-2045(24)00380-2 39116902

[B172] Rashid N. S. Grible J. M. Clevenger C. V. Harrell J. C. (2021). Breast cancer liver metastasis: current and future treatment approaches. Clin. and Exp. metastasis 38, 263–277. 10.1007/s10585-021-10080-4 33675501 PMC8211035

[B173] Rebecca L. S. Tyler B. K. Angela N G. Hyuna S. Ahmedin J. (2025). Cancer statistics, 2025. CA a cancer J. Clin. 75, 10–45. 10.3322/caac.21871

[B174] Rettig M. Weingarth M. Langel W. Kamal A. Kumar P. P. Weisz K. (2009). Solution structure of a covalently bound pyrrolo[2,1-c] [1,4]benzodiazepine-benzimidazole hybrid to a 10mer DNA duplex. Biochemistry 48, 12223–12232. 10.1021/bi901655t 19911838

[B175] Ricart A. D. (2011). Antibody-drug conjugates of calicheamicin derivative: Gemtuzumab ozogamicin and inotuzumab ozogamicin. Clin. Cancer Res. 17, 6417–6427. 10.1158/1078-0432.CCR-11-0486 22003069

[B176] Ríos-Luci C. García-Alonso S. Díaz-Rodríguez E. Nadal-Serrano M. Arribas J. Ocaña A. (2017). Resistance to the antibody-drug conjugate T-DM1 is based in a reduction in lysosomal proteolytic activity. Cancer Res. 77, 4639–4651. 10.1158/0008-5472.CAN-16-3127 28687619

[B177] Roos W. P. Kaina B. (2013). DNA damage-induced cell death: from specific DNA lesions to the DNA damage response and apoptosis. Cancer Lett. 332, 237–248. 10.1016/j.canlet.2012.01.007 22261329

[B178] Rosenberg J. E. O'Donnell P. H. Balar A. V. McGregor B. A. Heath E. I. Yu E. Y. (2019). Pivotal trial of enfortumab vedotin in urothelial carcinoma after platinum and anti-programmed death 1/Programmed death ligand 1 therapy. J. Clin. Oncol. 37, 2592–2600. 10.1200/JCO.19.01140 31356140 PMC6784850

[B179] Rugo H. S. Bardia A. Marmé F. Cortés J. Schmid P. Loirat D. (2023). Overall survival with sacituzumab govitecan in hormone receptor-positive and human epidermal growth factor receptor 2-negative metastatic breast cancer (TROPiCS-02): a randomised, open-label, multicentre, phase 3 trial. Lancet 402, 1423–1433. 10.1016/S0140-6736(23)01245-X 37633306

[B180] Sanderson R. J. Hering M. A. James S. F. Sun M. M. C. Doronina S. O. Siadak A. W. (2005). *In vivo* drug-linker stability of an anti-CD30 dipeptide-linked Auristatin immunoconjugate. Clin. Cancer Res. 11, 843–852. 10.1158/1078-0432.843.11.2 15701875

[B181] Sands J. Ahn M.-J. Lisberg A. Cho B. C. Blumenschein G. Shum E. (2025). Datopotamab deruxtecan in advanced or metastatic non-small cell lung cancer with actionable genomic alterations: results from the phase II TROPION-Lung05 study. J. Clin. Oncol. 43, 1254–1265. 10.1200/JCO-24-01349 39761483 PMC11949215

[B182] Santin A. D. Corr B. R. Spira A. Willmott L. Butrynski J. Tse K. Y. (2024). Efficacy and safety of sacituzumab govitecan in patients with advanced solid tumors (TROPiCS-03): analysis in patients with advanced endometrial cancer. J. Clin. Oncol. 42, 3421–3429. 10.1200/JCO.23.02767 39083724 PMC11458108

[B183] Sato K. Ando K. Okuyama S. Moriguchi S. Ogura T. Totoki S. (2018). Photoinduced ligand release from a silicon phthalocyanine dye conjugated with monoclonal antibodies: a mechanism of cancer cell cytotoxicity after near-infrared photoimmunotherapy. ACS Cent. Sci. 4, 1559–1569. 10.1021/acscentsci.8b00565 30555909 PMC6276043

[B184] Scaranti M. Cojocaru E. Banerjee S. Banerji U. (2020). Exploiting the folate receptor α in oncology. Nat. Rev. Clin. Oncol. 17, 349–359. 10.1038/s41571-020-0339-5 32152484

[B185] Sehn L. H. Salles G. (2021). Diffuse large B-Cell lymphoma. N. Engl. J. Med. 384, 842–858. 10.1056/NEJMra2027612 33657296 PMC8377611

[B186] Sehn L. H. Herrera A. F. Flowers C. R. Kamdar M. K. McMillan A. Hertzberg M. (2020). Polatuzumab vedotin in relapsed or refractory diffuse large B-Cell lymphoma. J. Clin. Oncol. 38, 155–165. 10.1200/jco.19.00172 31693429 PMC7032881

[B187] Sellner L. Fan F. Giesen N. Schubert M.-L. Goldschmidt H. Müller-Tidow C. (2020). B-cell maturation antigen-specific chimeric antigen receptor T cells for multiple myeloma: clinical experience and future perspectives. Int. J. Cancer 147, 2029–2041. 10.1002/ijc.33002 32270481

[B188] Shah N. N. Stevenson M. S. Yuan C. M. Richards K. Delbrook C. Kreitman R. J. (2015). Characterization of CD22 expression in acute lymphoblastic leukemia. Pediatr. Blood Cancer 62, 964–969. 10.1002/pbc.25410 25728039 PMC4405453

[B189] Shao Z. Pang D. Yang H. Li W. Wang S. Cui S. (2020). Efficacy, safety, and tolerability of pertuzumab, trastuzumab, and docetaxel for patients with early or locally advanced ERBB2-Positive breast cancer in Asia: the PEONY phase 3 randomized clinical trial. JAMA Oncol. 6, e193692. 10.1001/jamaoncol.2019.3692 31647503 PMC6813591

[B190] Sheng X. Yan X. Wang L. Shi Y. Yao X. Luo H. (2021). Open-label, multicenter, phase II study of RC48-ADC, a HER2-Targeting antibody-drug conjugate, in patients with locally advanced or metastatic urothelial carcinoma. Clin. Cancer Res. 27, 43–51. 10.1158/1078-0432.CCR-20-2488 33109737

[B191] Shi F. Liu Y. Zhou X. Shen P. Xue R. Zhang M. (2022). Disitamab vedotin: a novel antibody-drug conjugates for cancer therapy. Drug Deliv. 29, 1335–1344. 10.1080/10717544.2022.2069883 35506447 PMC9090390

[B192] Shin S. H. Ju E. J. Park J. Ko E. J. Kwon M. R. Lee H. W. (2023). ITC-6102RO, a novel B7-H3 antibody-drug conjugate, exhibits potent therapeutic effects against B7-H3 expressing solid tumors. Cancer Cell Int. 23, 172. 10.1186/s12935-023-02991-x 37596639 PMC10439577

[B193] Short N. J. Jabbour E. Jain N. Kantarjian H. (2024). Inotuzumab ozogamicin for the treatment of adult acute lymphoblastic leukemia: past progress, current research and future directions. J. Hematol. Oncol. 17, 32. 10.1186/s13045-024-01552-7 38734670 PMC11088766

[B194] Singh N. Frey N. V. Engels B. Barrett D. M. Shestova O. Ravikumar P. (2021). Antigen-independent activation enhances the efficacy of 4-1BB-costimulated CD22 CAR T cells. Nat. Med. 27, 842–850. 10.1038/s41591-021-01326-5 33888899 PMC8451032

[B195] Smit E. F. Felip E. Uprety D. Nagasaka M. Nakagawa K. Paz-Ares Rodríguez L. (2024). Trastuzumab deruxtecan in patients with metastatic non-small-cell lung cancer (DESTINY-Lung01): primary results of the HER2-overexpressing cohorts from a single-arm, phase 2 trial. Lancet Oncol. 25, 439–454. 10.1016/S1470-2045(24)00064-0 38547891

[B196] Staudacher A. H. Brown M. P. (2017). Antibody drug conjugates and bystander killing: is antigen-dependent internalisation required? Br. J. Cancer 117, 1736–1742. 10.1038/bjc.2017.367 29065110 PMC5729478

[B197] Stein H. Foss H. D. Dürkop H. Marafioti T. Delsol G. Pulford K. (2000). CD30(+) anaplastic large cell lymphoma: a review of its histopathologic, genetic, and clinical features. Blood 96, 3681–3695. 10.1182/blood.v96.12.3681 11090048

[B198] Stelljes M. Raffel S. Alakel N. Wäsch R. Kondakci M. Scholl S. (2024). Inotuzumab ozogamicin as induction therapy for patients older than 55 years with Philadelphia chromosome-negative B-Precursor ALL. J. Clin. Oncol. 42, 273–282. 10.1200/JCO.23.00546 37883727

[B199] Straus D. J. Długosz-Danecka M. Alekseev S. Illés Á. Picardi M. Lech-Maranda E. (2020). Brentuximab vedotin with chemotherapy for stage III/IV classical hodgkin lymphoma: 3-Year update of the ECHELON-1 study. Blood 135, 735–742. 10.1182/blood.2019003127 31945149

[B200] Strop P. Liu S. H. Dorywalska M. Delaria K. Dushin R. G. Tran T. T. (2013). Location matters: site of conjugation modulates stability and pharmacokinetics of antibody drug conjugates. Chem. Biol. 20, 161–167. 10.1016/j.chembiol.2013.01.010 23438745

[B201] Su Z. Xiao D. Xie F. Liu L. Wang Y. Fan S. (2021). Antibody–drug conjugates: recent advances in linker chemistry. Acta Pharm. Sin. B 11, 3889–3907. 10.1016/j.apsb.2021.03.042 35024314 PMC8727783

[B202] Sung M. Tan X. Lu B. Golas J. Hosselet C. Wang F. (2018). Caveolae-mediated endocytosis as a novel mechanism of resistance to trastuzumab emtansine (T-DM1). Mol. Cancer Ther. 17, 243–253. 10.1158/1535-7163.MCT-17-0403 29054985

[B203] Syed Y. Y. (2020). Sacituzumab govitecan: first approval. Drugs 80, 1019–1025. 10.1007/s40265-020-01337-5 32529410 PMC7288263

[B204] Takegawa N. Nonagase Y. Yonesaka K. Sakai K. Maenishi O. Ogitani Y. (2017). DS-8201a, a new HER2-targeting antibody-drug conjugate incorporating a novel DNA topoisomerase I inhibitor, overcomes HER2-positive gastric cancer T-DM1 resistance. Int. J. Cancer 141, 1682–1689. 10.1002/ijc.30870 28677116

[B205] Tamura K. Tsurutani J. Takahashi S. Iwata H. Krop I. E. Redfern C. (2019). Trastuzumab deruxtecan (DS-8201a) in patients with advanced HER2-positive breast cancer previously treated with trastuzumab emtansine: a dose-expansion, phase 1 study. Lancet Oncol. 20, 816–826. 10.1016/S1470-2045(19)30097-X 31047803

[B206] Tilly H. Morschhauser F. Sehn L. H. Friedberg J. W. Trněný M. Sharman J. P. (2022). Polatuzumab vedotin in previously untreated diffuse large B-Cell lymphoma. N. Engl. J. Med. 386, 351–363. 10.1056/NEJMoa2115304 34904799 PMC11702892

[B207] Tolaney S. M. Jiang Z. Zhang Q. Barroso-Sousa R. Park Y. H. Rimawi M. F. (2025a). Trastuzumab deruxtecan (T-DXd) + pertuzumab (P) vs taxane + trastuzumab + pertuzumab (THP) for first-line (1L) treatment of patients (pts) with human epidermal growth factor receptor 2–positive (HER2+) advanced/metastatic breast cancer (a/mBC): interim results from DESTINY-Breast09. J. Clin. Oncol. 43, LBA1008. 10.1200/JCO.2025.43.17_suppl.LBA1008

[B208] Tolaney S. M. Azambuja E. d. Kalinsky K. Loi S. Kim S.-B. Yam C. (2025b). Sacituzumab govitecan (SG) + pembrolizumab (pembro) vs chemotherapy (chemo) + pembro in previously untreated PD-L1–positive advanced triple-negative breast cancer (TNBC): primary results from the randomized phase 3 ASCENT-04/KEYNOTE-D19 study. J. Clin. Oncol. 43, LBA109. 10.1200/JCO.2025.43.17_suppl.LBA109

[B209] Tong J. H. Yeung S. F. Chan A. W. H. Chung L. Y. Chau S. L. Lung R. W. M. (2016). MET amplification and exon 14 splice site mutation define unique molecular subgroups of non-small cell lung carcinoma with poor prognosis. Clin. Cancer Res. 22, 3048–3056. 10.1158/1078-0432.CCR-15-2061 26847053

[B210] Tong J. T. W. Sarwar M. Ahangarpour M. Hume P. A. Williams G. M. Brimble M. A. (2024). Use of a cyclic α-Alkylidene-β-Diketone as a cleavable linker strategy for antibody-drug conjugates. J. Am. Chem. Soc. 146, 23717–23728. 10.1021/jacs.4c04567 39143910

[B211] Trudel S. Stewart A. K. (2024). A belantamab mafodotin revival in multiple myeloma therapy. N. Engl. J. Med. 391, 461–462. 10.1056/NEJMe2406799 39083777

[B212] Tsuchikama K. An Z. (2016). Antibody-drug conjugates: recent advances in conjugation and linker chemistries. Protein and Cell 9, 33–46. 10.1007/s13238-016-0323-0 27743348 PMC5777969

[B213] Uppal H. Doudement E. Mahapatra K. Darbonne W. C. Bumbaca D. Shen B.-Q. (2015). Potential mechanisms for thrombocytopenia development with trastuzumab emtansine (T-DM1). Clin. Cancer Res. 21, 123–133. 10.1158/1078-0432.CCR-14-2093 25370470

[B214] Urquhart L. (2019). FDA new drug approvals in Q2 2019. Nat. Rev. Drug Discov. 18, 575. 10.1038/d41573-019-00121-9 31367061

[B215] Vergote I. Van Nieuwenhuysen E. O'Cearbhaill R. E. Westermann A. Lorusso D. Ghamande S. (2023). Tisotumab vedotin in combination with carboplatin, pembrolizumab, or bevacizumab in recurrent or metastatic cervical cancer: results from the innovaTV 205/GOG-3024/ENGOT-cx8 study. J. Clin. Oncol. 41, 5536–5549. 10.1200/JCO.23.00720 37651655 PMC10730069

[B216] Vergote I. González-Martín A. Fujiwara K. Kalbacher E. Bagaméri A. Ghamande S. (2024). Tisotumab vedotin as Second- or third-line therapy for recurrent cervical cancer. N. Engl. J. Med. 391, 44–55. 10.1056/NEJMoa2313811 38959480

[B217] Verma S. Miles D. Gianni L. Krop I. E. Welslau M. Baselga J. (2012). Trastuzumab emtansine for HER2-positive advanced breast cancer. N. Engl. J. Med. 367, 1783–1791. 10.1056/NEJMoa1209124 23020162 PMC5125250

[B218] von Minckwitz G. Huang C.-S. Mano M. S. Loibl S. Mamounas E. P. Untch M. (2019). Trastuzumab emtansine for residual invasive HER2-Positive breast cancer. N. Engl. J. Med. 380, 617–628. 10.1056/NEJMoa1814017 30516102

[B219] Wang K. Wei G. Liu D. (2012). CD19: a biomarker for B cell development, lymphoma diagnosis and therapy. Exp. Hematol. Oncol. 1, 36. 10.1186/2162-3619-1-36 23210908 PMC3520838

[B220] Wang J. Anderson M. G. Oleksijew A. Vaidya K. S. Boghaert E. R. Tucker L. (2017). ABBV-399, a c-Met antibody-drug conjugate that targets both MET-amplified and c-Met-Overexpressing tumors, irrespective of MET pathway dependence. Clin. Cancer Res. 23, 992–1000. 10.1158/1078-0432.CCR-16-1568 27573171

[B221] Wang J. Li C. He K. Kuang Z. Lu J. Yao Y. (2023). Characterization of anti-CD79b/CD3 bispecific antibody, a potential therapy for B cell malignancies. Cancer Immunol. Immunother. 72, 493–507. 10.1007/s00262-022-03267-5 35963895 PMC10992295

[B222] Wei B. Gunzner-Toste J. Yao H. Wang T. Wang J. Xu Z. (2018). Discovery of peptidomimetic antibody-drug conjugate linkers with enhanced protease specificity. J. Med. Chem. 61, 989–1000. 10.1021/acs.jmedchem.7b01430 29227683

[B223] Wells G. Martin C. R. H. Howard P. W. Sands Z. A. Laughton C. A. Tiberghien A. (2006). Design, synthesis, and biophysical and biological evaluation of a series of pyrrolobenzodiazepine-poly(N-methylpyrrole) conjugates. J. Med. Chem. 49, 5442–5461. 10.1021/jm051199z 16942018

[B224] Xi M. Zhu J. Zhang F. Shen H. Chen J. Xiao Z. (2024). Antibody-drug conjugates for targeted cancer therapy: recent advances in potential payloads. Eur. J. Med. Chem. 276, 116709. 10.1016/j.ejmech.2024.116709 39068862

[B225] Xu B. Ma F. Wang T. Wang S. Tong Z. Li W. (2023). A phase IIb, single arm, multicenter trial of sacituzumab govitecan in Chinese patients with metastatic triple-negative breast cancer who received at least two prior treatments. Int. J. Cancer 152, 2134–2144. 10.1002/ijc.34424 36621000

[B226] Xu B. Yin Y. Fan Y. Ouyang Q. Song L. Wang X. (2024). Sacituzumab tirumotecan (SKB264/MK-2870) in patients (pts) with previously treated locally recurrent or metastatic triple-negative breast cancer (TNBC): results from the phase III OptiTROP-Breast01 study. J. Clin. Oncol. 42, 104. 10.1200/JCO.2024.42.16_suppl.104

[B227] Xue T. Xiao L. Liu Q. Song S. Shan A. Zhang X. (2024). Abstract 4702: medilink’s TMALIN ADC linker technology: tumor microenvironment specific extracellular and intracellular double cleavage mechanism for better efficacy and expanded target space. Cancer Res. 84, 4702. 10.1158/1538-7445.Am2024-4702

[B228] Yaghoubi S. Gharibi T. Karimi M. H. Sadeqi Nezhad M. Seifalian A. Tavakkol R. (2021). Development and biological assessment of MMAE-Trastuzumab antibody-drug conjugates (ADCs). Breast Cancer 28, 216–225. 10.1007/s12282-020-01153-5 32889587

[B229] Yagisawa M. Taniguchi H. Satoh T. Kadowaki S. Sunakawa Y. Nishina T. (2024). Trastuzumab deruxtecan in advanced solid tumors with human epidermal growth factor receptor 2 amplification identified by plasma cell-free DNA testing: a multicenter, single-arm, phase II basket trial. J. Clin. Oncol. 42, 3817–3825. 10.1200/jco.23.02626 39088783 PMC11542975

[B230] Yang Y. Zhang L. Ma Y. Zhao Y. Fang W. Zhao H. (2025). Phase I study of iza-bren (BL-B01D1), an EGFR x HER3 bispecific antibody-drug conjugate (ADC), in patients with locally advanced or metastatic non-small cell lung cancer (NSCLC) with driver genomic alterations (GA) outside of classic EGFR mutations. J. Clin. Oncol. 43, 3001. 10.1200/JCO.2025.43.16_suppl.3001

[B231] Yao X. Jiang J. Wang X. Huang C. Li D. Xie K. (2015). A novel humanized anti-HER2 antibody conjugated with MMAE exerts potent anti-tumor activity. Breast Cancer Res. Treat. 153, 123–133. 10.1007/s10549-015-3503-3 26253944

[B232] Yao H. Yan M. Tong Z. Wu X. Ryu M.-H. Park J. J. (2024). Safety, efficacy, and pharmacokinetics of SHR-A1811, a human epidermal growth factor receptor 2-Directed antibody-drug conjugate, in human epidermal growth factor receptor 2-Expressing or mutated advanced solid tumors: a global phase I trial. J. Clin. Oncol. 42, 3453–3465. 10.1200/JCO.23.02044 38900984

[B233] Ye D. Jiang S. Yuan F. Zhou F. Jiang K. Zhang X. (2025). Efficacy and safety of sacituzumab Tirumotecan monotherapy in patients with advanced urothelial carcinoma who progressed on or after prior anti-cancer therapies: report from the phase 1/2 MK-2870-001 study. J. Clin. Oncol. 43, 796. 10.1200/JCO.2025.43.5_suppl.796

[B234] Yin Y. Fan Y. Ouyang Q. Song L. Wang X. Li W. (2025). Sacituzumab tirumotecan in previously treated metastatic triple-negative breast cancer: a randomized phase 3 trial. Nat. Med. 31, 1969–1975. 10.1038/s41591-025-03630-w 40217078

[B235] Younes A. Gopal A. K. Smith S. E. Ansell S. M. Rosenblatt J. D. Savage K. J. (2012a). Results of a pivotal phase II study of brentuximab vedotin for patients with relapsed or refractory hodgkin's lymphoma. J. Clin. Oncol. 30, 2183–2189. 10.1200/JCO.2011.38.0410 22454421 PMC3646316

[B236] Younes A. Gopal A. K. Smith S. E. Ansell S. M. Rosenblatt J. D. Savage K. J. (2012b). Results of a pivotal phase II study of brentuximab vedotin for patients with relapsed or refractory hodgkin's lymphoma. J. Clin. Oncol. 30, 2183–2189. 10.1200/jco.2011.38.0410 22454421 PMC3646316

[B237] Yuen S. Phillips T. J. Bannerji R. Marlton P. Gritti G. Seymour J. F. (2024). Polatuzumab vedotin, venetoclax, and an anti-CD20 monoclonal antibody in relapsed/refractory B-cell Non-Hodgkin lymphoma. Am. J. Hematol. 99, 1281–1289. 10.1002/ajh.27341 38700035

[B238] Zammarchi F. Corbett S. Adams L. Tyrer P. C. Kiakos K. Janghra N. (2018). ADCT-402, a PBD dimer-containing antibody drug conjugate targeting CD19-expressing malignancies. Blood 131, 1094–1105. 10.1182/blood-2017-10-813493 29298756

[B239] Zhang T. You L. Xu J. Yin J. Qu B. Mao Y. (2023). Abstract LB031: SHR-A1811, a novel anti-HER2 ADC with superior bystander effect, optimal DAR and favorable safety profiles. Cancer Res. 83, LB031. 10.1158/1538-7445.Am2023-lb031

[B240] Zhao X. Cheng C. Gou J. Yi T. Qian Y. Du X. (2018). Expression of tissue factor in human cervical carcinoma tissue. Exp. Ther. Med. 16, 4075–4081. 10.3892/etm.2018.6723 30402151 PMC6200962

[B241] Zhou K. I. Strickler J. H. Chen H. (2024). Targeting Claudin-18.2 for cancer therapy: updates from 2024 ASCO annual meeting. J. Hematol. Oncol. 17, 73. 10.1186/s13045-024-01595-w 39183320 PMC11346204

[B242] Zhou L. Yang K. W. Zhang S. Yan X. Q. Li S. M. Xu H. Y. (2025). Disitamab vedotin plus toripalimab in patients with locally advanced or metastatic urothelial carcinoma (RC48-C014): a phase Ib/II dose-escalation and dose-expansion study. Ann. Oncol. 36, 331–339. 10.1016/j.annonc.2024.12.002 39662628

[B243] Zhu Y. Zhu X. Wei X. Tang C. Zhang W. (2021). HER2-targeted therapies in gastric cancer. Biochim. Biophys. Acta Rev. Cancer 1876, 188549. 10.1016/j.bbcan.2021.188549 33894300

