# Correction: Antibody-drug conjugates in cancer therapy: current advances and prospects for breakthroughs

**DOI:** 10.3389/fcell.2026.1860706

**Published:** 2026-05-25

**Authors:** Dan Wu, Kaixuan Yang, Runjia He, Rutie Yin, Lin Shui

**Affiliations:** 1 Cancer Center, West China Second University Hospital, Sichuan University, Chengdu, Sichuan, China; 2 Key Laboratory of Birth Defects and Related Diseases of Women and Children, Sichuan University, Chengdu, Sichuan, China; 3 School of Pharmacy, Southwest Medical University, Luzhou, Sichuan, China; 4 Acupuncture and Tuina School, Chengdu University of Traditional Chinese Medicine, Chengdu, Sichuan, China

**Keywords:** antibody drug conjugate (ADC), antitumor treatment, linker, payload, drug approval

There was a mistake in Figure 4 as published. MP binds to CD22 instead of CD19. The corrected Figure 4 appears below.

**FIGURE 4:**
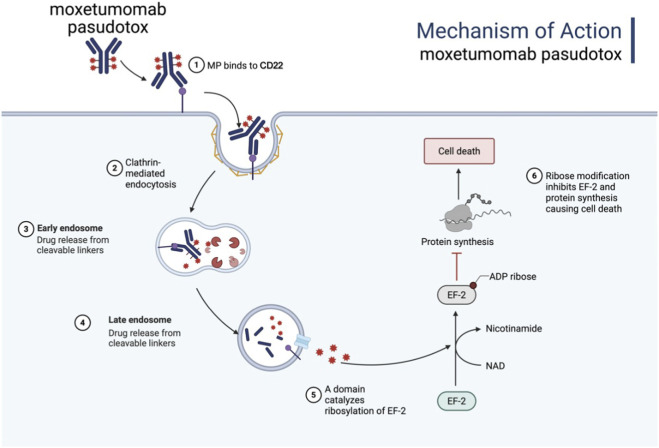
The mechanism of action of Moxetumomab pasudotox. It is internalized into the cell *via* endocytosis, forming an endosome. Within the acidic environment of the endosome, the immunotoxin releases the PE38 toxin, which inhibits protein synthesis by targeting EF-2, ultimately inducing apoptosis or necrosis in tumor cells. Figure was created by Biorender.com.

The funder Natural Science Foundation of Sichuan Province for the general programs (No. 2024NSFSC1877) to Kaixuan Yang was erroneously omitted.

The original article has been updated.

